# SSVEP detection assessment by combining visual stimuli paradigms and no-training detection methods

**DOI:** 10.3389/fnins.2023.1142892

**Published:** 2023-05-18

**Authors:** Juan David Chailloux Peguero, Luis G. Hernández-Rojas, Omar Mendoza-Montoya, Ricardo Caraza, Javier M. Antelis

**Affiliations:** ^1^Tecnologico de Monterrey, School of Engineering and Sciences, Monterrey, Mexico; ^2^Tecnologico de Monterrey, School of Medicine and Health Sciences, Monterrey, Mexico

**Keywords:** SSVEP detection method, SSVEP visual paradigm, BCI-user comfort, Brain-Computer Interface, electroencephalography, evoked potentials, biomedical signal processing

## Abstract

**Introduction:**

Brain-Computer Interfaces (BCI) based on Steady-State Visually Evoked Potentials (SSVEP) have great potential for use in communication applications because of their relatively simple assembly and in some cases the possibility of bypassing the time-consuming training stage. However, among multiple factors, the efficient performance of this technology is highly dependent on the stimulation paradigm applied in combination with the SSVEP detection algorithm employed. This paper proposes the performance assessment of the classification of target events with respect to non-target events by applying four types of visual paradigms, rectangular modulated On-Off (OOR), sinusoidal modulated On-Off (OOS), rectangular modulated Checkerboard (CBR), and sinusoidal modulated Checkerboard (CBS), with three types of SSVEP detection methods, Canonical Correlation Analysis (CCA), Filter-Bank CCA (FBCCA), and Minimum Energy Combination (MEC).

**Methods:**

We set up an experimental protocol in which the four types of visual stimuli were presented randomly to twenty-seven participants and after acquiring their electroencephalographic responses to five stimulation frequencies (8.57, 10.909, 15, 20, and 24 Hz), the three detection methods were applied to the collected data.

**Results:**

The results are conclusive, obtaining the best performance with the combination of either OOR or OOS visual stimulus and the FBCCA as a detection method, however, this finding contrasts with the opinion of almost half of the participants in terms of visual comfort, where the 51.9% of the subjects felt more comfortable and focused with CBR or CBS stimulation.

**Discussion:**

Finally, the EEG recordings correspond to the SSVEP response of 27 subjects to four visual paradigms when selecting five items on a screen, which is useful in BCI navigation applications. The dataset is available to anyone interested in studying and evaluating signal processing and machine-learning algorithms for SSVEP-BCI systems.

## 1. Introduction

The phenomenon of Steady-State Visual Evoked Potentials (SSVEP) is manifested as electrical brain patterns elicited when a user focuses his/her attention on a repetitive visual stimulus (a light source) flickering at frequencies higher than 6 Hz (Faller et al., 2017). These are periodic oscillations prominently observed in the occipital and occipito-parietal areas of the cerebral cortex. SSVEP responses appear as an increase in the amplitude of the signal at the fundamental frequency and its harmonics for the corresponding stimulus attended by the user (Antelis et al., 2020). In addition to the usual clinical purpose of diagnosing visual pathway and brain mapping impairments, the SSVEP can serve as a basis for Brain-Computer Interfaces (BCI) applications (Amiri et al., 2013; Chen et al., 2021).

BCI can be considered systems within the field of biomedical engineering and neurotechnologies, with the role of restoring or replacing lost neurological functions (Bockbrader et al., 2018), control of devices (Velasco-Álvarez et al., 2021) and establishing communication channels by alternative mechanisms, applying digital signal processing and machine learning techniques to electrical brain waves (McFarland et al., 1997). These technologies use different paradigms that allow them to infer mental states. The main paradigms we can find are: P300 (Farwell and Donchin, 1988), motor imagery (Lotze and Cohen, 2006; Jiang et al., 2020; Pei et al., 2022), and SSVEP (Volosyak et al., 2009). BCIs based on the SSVEP paradigm are the most widespread (Singla, 2018) for the sake of high communication rate, easy system configuration, and less user training (Gao et al., 2003, 2014; Wang et al., 2008; Chen et al., 2015b).

Currently, it is still a pending issue to implement SSVEP-based BCI applications with optimum performance that also meets the user's comfort requirements. The reason for this is due to the multi-factor dependence of SSVEP-based BCI on several elements to achieve the best execution. In response to this, Li et al. (2021) identified four ways to optimize BCI systems based on the SSVEP phenomenon used with spelling function: improving the classification algorithm, adding a spelling prediction function, designing better stimulation paradigms, and adding new triggering methods. The number of target elements, which can be considered as one of the most sensitive (Gembler et al., 2016) parameters, have a direct influence on each of the four optimization methods mentioned. The design of the stimuli, previously noted, is another key aspect since different associated properties are considered, such as the frequency at which the stimuli oscillate, the dimensions of the stimuli, the colors presented, and the type of signal that controls the stimuli (Cysewska-Sobusiak and Jukiewicz, 2016). In the study carried out by Siribunyaphat and Punsawad (2022), an exhaustive analysis of various visual stimulation proposals for BCIs based on the SSVEP phenomenon was performed. This paper reported a summary of the state of the art regarding the design of visual stimuli in SSVEP-based BCI, taking into account parameters such as the Proposed Method, which was included to evaluate performance, the Visual Stimulus, the Electrodes Position, and the Results obtained. All this with the objective of exploring alternative visual paradigms that would achieve better visual comfort and at the same time guarantee a good performance of the BCI. SSVEP detection methods are undoubtedly another aspect to be taken into account for the proper performance of the BCI.

When the visual paradigm is discussed, the type of signal that modulates the flicker is rarely considered. Generally the waveforms used are triangular, sinusoidal, and rectangular, the latter with different levels of duty cycle. According to relevant studies (Teng et al., 2011; Chen et al., 2019), modulation of the stimulus with a rectangular signal leads to better performance results. The waveform that modulates the luminance of the SSVEP stimulation signal has been the subject of relatively few studies compared to other parameters of the visual stimulus, such as the number of targets or the detection methods. Although the answer to the fact that there are so few studies addressing the effect on the performance based on the waveform of the modulating signal may lie in the minor importance of this aspect, it is pertinent to argue that the choice of this parameter of the visual stimulus influences the number of targets to be presented.

Results reported in Li et al. (2021) and Siribunyaphat and Punsawad (2022) compiles several published studies where the number of targets, the design of the stimulus, the SSVEP detection methods, the electrode position, number of subjects, frequencies of stimuli, and the results obtained are taken into account. Although the multi-factorial influence of these parameters is well-known, most studies address their association with performance individually rather than comprehensively. In addition, the combination of parameters that makes optimal the BCI performance is particular to each user, which makes necessary a previous process of calibration.

Newly implemented SSVEP detection algorithms require databases to evaluate their effectiveness. Similarly, certain BCI applications based on the SSVEP paradigm often rely on databases to estimate their viability (Bian et al., 2022). However, with respect to other paradigms, such as motor imagery (Pei et al., 2021) and P300, the SSVEP databases are scarce, as has been reported by Choi et al. (2019a). In the SSVEP database supplied by İşcan and Nikulin (2018) a four-class SSVEP-based BCI was assessed under different perturbations, where the subjects were speaking, thinking, or listening depending on the given task. Liu et al. (2022) implemented an SSVEP database targeting the elderly population, this way providing an opportunity to design BCI systems better suited for eldercare applications. Zhu et al. (2021) makes an interesting contribution by proposing an open-access dataset with a large number of subjects (102) for a wearable SSVEP-based BCI toward practical applications. This proposal comprehensively compares the SSVEP data obtained by wireless wet and dry electrodes. A concise and detailed analysis of the availability of SSVEP databases and the need for public domain access to them was carried out at Liu et al. (2020), concluding that there is a demand for more SSVEP paradigm databases to foster method design and evaluation.

Based on the literature reviewed, we want to answer the question of whether there is a specific combination of SSVEP's visual stimulus scheme with a no-training detection method that arouses a better performance in terms of Accuracy (ACC) and Information Transfer Rate (ITR) when classifying target events with respect to non-target events. According to Li et al. (2021), no study to date has compared the available paradigms to identify the one that delivers the best performance because, when the same methods of SSVEP detection are used, the results obtained are inconsistent owing to the different paradigms used, and thus the influence of the paradigm on the performance of the SSVEP speller cannot be ignored. We addressed this challenge by comparing four types of visual paradigms, i.e., rectangular modulated On-Off (OOR), sinusoidal modulated On-Off (OOS), rectangular modulated Checkerboard (CBR), and sinusoidal modulated Checkerboard (CBS), and studied their interaction with three SSVEP detection methods, i.e., Canonical Correlation Analysis (CCA), Filter-Bank CCA (FBCCA), and Minimum Energy Combination (MEC). We conducted an experiment where we captured non-invasive electroencephalographic (EEG) signals from 27 participants. These subjects were exposed to the four types of visual stimuli, distributed in 4 rounds of 10 min, which at the same time were subdivided into 40 trials of 15 s organized in 5 s of pre-stimulus, 5 s of stimulation, and 5 s of rest. The acquired brain signals were processed and transformed to the frequency domain, where the influence of the type of visual stimulus on the spectral power was evaluated. At the same time, the three SSVEP detection methods were applied to the electroencephalographic dataset, obtaining results in terms of ACC and ITR, where we analyzed which combination (visual stimulus type and detection method) achieved the best performance. At the time this research was conceived, we were unable to find studies that addressed in an integrated manner the influence of parameters such as the visual stimulus paradigm and the SSVEP detection method on the performance when classifying target events with respect to non-target events.

The novelty of our proposal lies in the integrated analysis of two aspects that have a direct impact on the performance of SSVEP-based BCI systems, specifically the visual paradigm applied and the detection method employed. Finally, another contribution of our study lies in the electroencephalographic dataset obtained during the experimental sessions. These training datasets correspond to the SSVEP response to four visual paradigms in which 27 subjects were shown five flashing elements on an LCD screen. This provides approximately 1,080 min of EEG. Our database is not oriented to spelling applications, as is the case with most publicly available SSVEP databases. It is rather oriented as a complement for the assessment of SSVEP detection algorithms and navigation applications although it could have multi-stage communication applications, where the graphical interface has two or more stages and the number of targets in each stage is relatively small (Li et al., 2021).

Our work is structured in a Section 1; a Section 2, where the experimental protocol is described, and the analyses performed on the EEG signals are applied; a Section 3, where we report the results obtained from applying different visual stimuli and SSVEP detection methods; a Section 4, where the results obtained are analyzed and contrasted with the literature consulted, and finally, a Section 5, where the main findings of the research are summarized and future studies derived from this research are suggested.

## 2. Methods and materials

### 2.1. Visual stimulus types

In this work, we consider the on-off and checkerboard patterns with luminance modulated by rectangular and sinusoidal functions, resulting in a total of four types of visual stimuli. The description of these visual stimuli is as follows:

- *On-Off pattern with rectangular modulated signal (OOR)*. The luminance in this type of visual stimulus switches suddenly and repeatedly with each rising and falling edge following a rectangular function. High and low states of the function indicate that the visual stimulus is fully on and fully off, respectively, therefore, there are no intermediate intensities for the visual stimulus.
- *On-Off pattern with sinusoidal modulated signal (OOS)*. In this case, the luminance of the visual stimulus gradually turns on and off according to a sine function. Crests and valleys represent the visual stimulus fully on and fully off, respectively, while the rest of the sinusoidal shape represents intermediate intensities of the visual stimulus.
- *Checkerboard pattern with rectangular modulated signal (CBR)*. This visual stimulus consists of a matrix (usually 8 × 8, but can vary) with squares whose luminance alternates following a rectangular function. The transition from high to low (and vice versa) in the rectangular function indicates the sudden luminance change of the boxes in the visual stimulus.
- *Checkerboard pattern with sinusoidal modulated signal (CBS)*. In this case, the luminance of the squares in the matrix gradually varies according to a sinusoidal function. Crests/valleys represent the visual stimulus with squares fully on/off and the rest of the sinusoidal shape represents intermediate color intensities of the visual stimulus.

**Figure 1:**
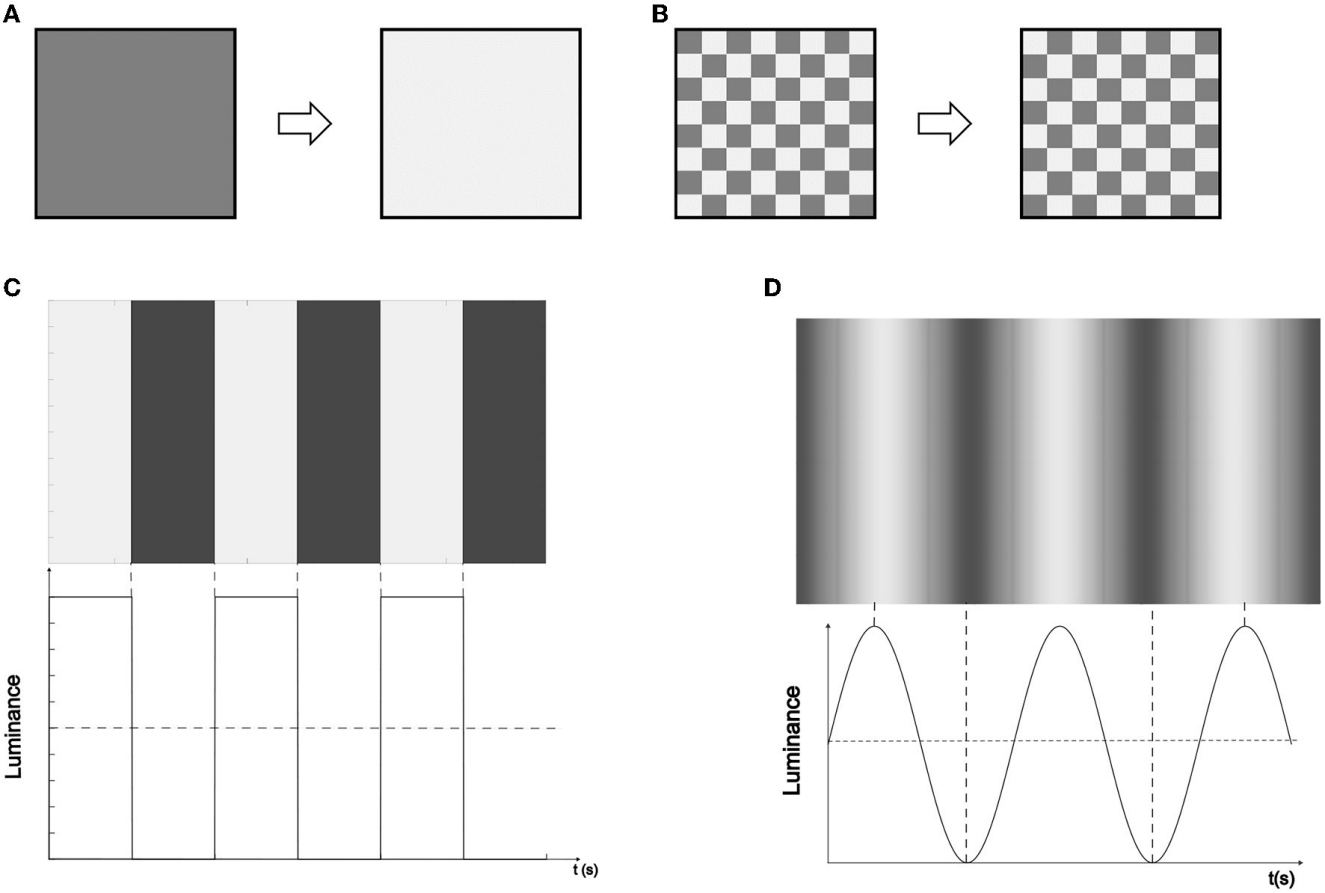
Illustration of how the intensity of the On-Off and Checkerboard patterns are modulated by rectangular and sinusoidal functions. **(A)** On-Off paradigm transition, where the square's luminance alternates with the background controlled by a modulating periodic waveform. **(B)** Checkerboard paradigm transition, where the luminance of the square grid array alternates controlled by a modulating periodic waveform. **(C)** Rectangular waveform luminance modulation. **(D)** Sine waveform luminance modulation.

We choose the on-off and checkerboard patterns because these two types of stimuli have generally been used to evoke SSVEPs for a BCI application on computer screens (Zhu et al., 2010), and the rectangular and sinusoidal modulated signals because these waveforms are the most employed to control the stimulus luminance (Cysewska-Sobusiak and Jukiewicz, 2016; Chen et al., 2019). Note that for the case of visual stimulus based on the on-off pattern the frequency of the modulating signal represents the stimulation frequency expected to be observed in the SSVEP responses. However, for visual stimulus based on the checkerboard pattern, the stimulation frequency expected to be observed in the SSVEP responses is twice the frequency of the modulating signal. This is because the SSVEP is produced at its rate of phase-reversal or alternation rate (Regan, 1977; Burkitt et al., 2000; Lalor et al., 2005). As an illustration, Figure 1 depicts how the intensity of the on-off pattern is modulated by rectangular and sinusoidal functions. In this work, the colors of the four types of visual stimulus were black and white with color transitions from black, in the off-state, to white, in the on-state.

#### 2.2. SSVEP detection methods

The SSVEP detection methods employed have the particularity that they do not require prior training to be applied to EEG recordings. To present the technical details, we define a multidimensional variable **X** = [_*x*_*e*_(*t*)]*N*_*e*_×*N*_*t*__ that is representative of the brain electrical activity captured on the scalp, organized in segments of *N*_*e*_ electrodes and *N*_*t*_ samples.

It is assumed at all times that the analyzed signals correspond to the instances in which the participants were gazing at each blinking element applied during an experimental session, thus corresponding to EEG recordings in which the participant focused on one of the *N*_*target*_ visual stimuli that were flickering at a frequency *f*_*i*_∈ℝ; *f*_*i*_:{*f*_1_, *f*_2_, ..., *f*_*N*_*target*__}. In each of the approaches, the goal is to estimate which of the targets the participant was focusing on considering as cues the EEG activity **X**, the *N*_*target*_ sources of visual stimulation, and the *f*_*i*_ stimulation frequencies.

#### 2.2.1. Canonical correlation analysis (CCA)

In the SSVEP CCA method, the idea is to find linear combinations that maximize the correlation between two vectors: the EEG signal samples **X** = [_*x*_*e*_(*t*)]*N*_*e*_×*N*_*t*__ corresponding to the moment when the subject is gazing at one of the *N*_*target*_ blinking targets; and a template array of reference sinusoidal signals: **Y_f_i__** = [_*y*_*r*_(*t*)]2*N*_*h*_×*N*_*t*__ for each of the *f*_*i*_ stimulation frequencies, where *N*_*h*_ represents the number of harmonics for a sinusoidal template signal of frequency *f*_*i*_. Here we define **Y_f_i__** as:


(1) $$Y_{f_{i}}(t)=\left[\begin{array}{c} sin(2\pi f_{i}t)\\ cos(2\pi f_{i}t)\\ \vdots \\ sin(2\pi N_{h}f_{i}t)\\ cos(2\pi N_{h}f_{i}t) \end{array}\right]$$

The linear combinations are defined as **p** = **Xw**_*p*_ and **q** = **Y_f_i__w**_*q*_ that maximize the so-called canonical correlation ρ between them. Hence, the weight vectors ${\text{w}}_{p}\in {\mathbb{R}}^{N_{e}\times 1}$ and ${\text{w}}_{q}\in {\mathbb{R}}^{2N_{h}\times 1}$ are found by solving:


(2) $$\begin{array}{l} \rho =\underset{{\text{w}}_{p},{\text{w}}_{q}}{\max}corr\left(p,\text{q}\right) \end{array}$$

which can be rewritten as the following optimization problem (Zhang et al., 2014):


(3) $$\begin{array}{l} \rho =\underset{{\text{w}}_{p},{\text{w}}_{q}}{\max}\frac{{\text{w}}_{p}^{⊤}{\text{C}}_{pq}{\text{w}}_{q}}{\sqrt{{\text{w}}_{p}^{⊤}{\text{C}}_{pp}{\text{w}}_{p}{\text{w}}_{q}^{⊤}{\text{C}}_{qq}{\text{w}}_{q}}} \end{array}$$

where **C**_*pq*_ is the cross-covariance matrix and **C**_*pp*_ and **C**_*qq*_ are the auto-covariance matrices for **X** and **Y_f_i__**, respectively. The solution is obtained by solving a generalized eigenvalue problem (Hardoon et al., 2004), from which the weight vector **w**_*p*_ is an eigenvector of ${\text{C}}_{pp}^{-1}{\text{C}}_{pq}{\text{C}}_{qq}^{-1}{\text{C}}_{qp}$ and the weight vector **w**_*q*_ is an eigenvector of ${\text{C}}_{qq}^{-1}{\text{C}}_{qp}{\text{C}}_{pp}^{-1}{\text{C}}_{pq}$. The maximum canonical correlation corresponds to the maximum value of ρ with respect to **w**_*q*_ and **w**_*p*_ (Hardoon et al., 2004).

#### 2.2.2. Filter-bank canonical correlation analysis (FBCCA)

The ability to incorporate harmonic components in frequency identification methods is an advantage since they provide useful information for the performance of these procedures. The FBCCA method is able to extract the discriminative information embedded in the harmonic components of the SSVEP responses in a more efficient way. This is achieved by using band-pass filter banks that decompose the SSVEPs into several sub-band components. By splitting the original SSVEP response into several signal versions with different bandwidths, it is ensured that not only the fundamental frequency component contributes to the detection process but also its different harmonics. FBCCA method was proposed to enhance the CCA detection method on SSVEP phenomenon (Chen et al., 2015a). The algorithm involves three stages: filter bank analysis; CCA between the SSVEP components filtered by sub-bands and the reference sinusoidal signals, and finally; the identification of the target element.

First, a filter bank analysis, with *SB* band-pass filters, was applied on the EEG segment **X** and from this procedure, *SB* versions of the original signal were obtained (**X_j_**, *j* = 1, 2, ..., *SB*). In our study, five Chebyshev band-pass type I Infinite Impulse Response (IIR) filters were implemented with pass bands ranging from 6 − 90, 9 − 90, 13 − 90, 18 − 90, and 22 − 90 Hz, respectively (Chen et al., 2015a). CCA was then applied between the reference signal (**Y_f_i__**, *i* = 1, 2, ..., *N*_*target*_) and each **X_j_** sub-band component separately:


(4) $$\rho_{f_{i}}=\left[\begin{array}{c} \rho_{f_{i}}^{1}\\ \;\\ \rho_{f_{i}}^{2}\\ \;\\ \vdots \\ \;\\ \rho_{f_{i}}^{N_{SB}} \end{array}\right]$$

(5) $$\begin{array}{l} =\left[\begin{array}{c} \rho \left({\text{X}}_{j_{1}}^{⊤}{\text{W}}_{\text{X}}\left({\text{X}}_{j_{1}}{\text{Y}}_{f_{i}}\right),{\text{Y}}^{⊤}{\text{W}}_{\text{Y}}\left({\text{X}}_{j_{1}}{\text{Y}}_{f_{i}}\right)\right)\\ \rho \left({\text{X}}_{j_{2}}^{⊤}{\text{W}}_{\text{X}}\left({\text{X}}_{j_{2}}{\text{Y}}_{f_{i}}\right),{\text{Y}}^{⊤}{\text{W}}_{\text{Y}}\left({\text{X}}_{j_{2}}{\text{Y}}_{f_{i}}\right)\right)\\ \vdots \\ \rho \left({\text{X}}_{j_{SB}}^{⊤}{\text{W}}_{\text{X}}\left({\text{X}}_{j_{SB}}{\text{Y}}_{f_{i}}\right),{\text{Y}}^{⊤}{\text{W}}_{\text{Y}}\left({\text{X}}_{j_{SB}}{\text{Y}}_{f_{i}}\right)\right) \end{array}\right] \end{array}$$

Sub-band EEG components were obtained for each frequency by applying a weighted sum of squares of the previously derived coefficients:


(6) $$\begin{array}{l} {\tilde{\rho }}_{f_{i}}=\overset{SB}{\underset{n=1}{\sum }}w\left(n\right)\cdot {\left(\rho_{f_{i}}^{n}\right)}^{2} \end{array}$$

In Equation (6), *n* is the sub-band index.

The target frequency is then considered to correspond to the reference signal yielding the maximal ${\tilde{\rho }}_{f_{i}}$.

In our proposal, we used the fundamental frequency and three harmonics of this, for a total of *N*_*h*_ = 4. We chose this number of harmonics since it was the one that gave the best performance when testing different values from 1 to 10 with the standard CCA method, as proposed in Chen et al. (2015a).

#### 2.2.3. Minimum energy combination (MEC)

The Minimum Energy Combination finds spatial filters to improve EEG responses of the oscillatory components modulated in one particular control task (Friman et al., 2007). An EEG signal segment can be modeled as follows:


(7) $$\begin{array}{l} x_{e}\left(t\right)=\overset{N_{h}}{\underset{h=1}{\sum }}\left(a_{e,h}sin\left(2\pi hf_{i}t\right)+b_{e,h}cos\left(2\pi hf_{i}t\right)\right)+\eta_{e,f_{i}}\left(t\right) \end{array}$$

here *N*_*h*_ (as in CCA) is the number of harmonics of the model; *a*_*e, h*_ and *b*_*e, h*_ are multiplicative constants for each channel *e* and harmonic *h* and η_*e*,_*f*__*i*__(*t*) represents the measured activity that is not associated with the SSVEP manifestation. This relationship can also be expressed in matrix format as follows (Volosyak, 2011):


(8) $$\begin{array}{l} \text{X}=\text{G}{\text{X}}_{f_{i}}^{ref}+\eta_{f_{i}} \end{array}$$

In Equation (8), **G** is defined as


(9) $$G={\left|\begin{array}{ccccc} a_{1,1} & b_{1,1} & ... & a_{1,n_{h}} & b_{1,N_{h}}\\ a_{2,1} & b_{2,2} & ... & a_{2,n_{h}} & b_{2,N_{h}}\\ \vdots & \vdots & ... & \vdots & \vdots \\ a_{N_{e},1} & b_{N_{e},1} & ... & a_{N_{e},N_{h}} & b_{N_{e},N_{h}} \end{array}\right|}_{N_{e}\times 2N_{h}}$$

where *N*_*e*_ represents the number of channels and **η**_*f*_*i*__ = [_η_*e*,_*f*__*i*__(*t*)]*N*_*e*_×*N*_*t*__.

An optimized approximation of the **η**_*f*_*i*__ matrix can be obtained from Equation (8)


(10) $$\eta_{f_{i}}^{*}=X-G^{*}X_{f_{i}}^{ref}$$

As $\eta_{f_{i}}^{*}$ represents the interfering and artifact activity captured at the electrodes, then the objective is to minimize this manifestation. The MEC method precisely obtains *N*_*m*_ spatial filters that minimize the energy of $\eta_{f_{i}}^{*}$ in such a way that the new channels obtained only contain information associated with the brain's electrical activity resulting from the visual stimulation paradigm applied at a given frequency *f*_*i*_. The matrix of spatial filters **W** is obtained from


(11) $$\begin{array}{l} \text{W}=\left[\frac{v_{1}}{\sqrt{\lambda_{1}}}\frac{v_{2}}{\sqrt{\lambda_{2}}}...\frac{v_{N_{m}}}{\sqrt{\lambda_{N_{m}}}}\right]=\left[w^{1}w^{2}...w^{N_{m}}\right] \end{array}$$

where *N*_*m*_ is the number of spatial filters; *v*_*i*_ = {*v*_1_, *v*_2_, ..., *v*_*N*_*m*__} and λ_*i*_ = {λ_1_, λ_2_, ..., λ_*N*_*m*__} are the eigenvectors and their corresponding eigenvalues for the optimized matrix **M** defined by $\text{M}=\eta_{f}^{*}{\left(\eta_{f}^{*}\right)}^{⊤}$.

Finally, the average power for each target frequency *f*_*i*_ is obtained as


(12) $$\begin{array}{l} p_{f_{i}}=\frac{1}{N_{m}N_{h}}\overset{N_{m}}{\underset{i=1}{\sum }}\overset{2N_{h}}{\underset{j=1}{\sum }}\overset{N_{t}}{\underset{t=1}{\sum }}\left(x_{j,f_{i}}^{ref}\left(t\right)\overset{N_{e}}{\underset{e=1}{\sum }}w_{e}^{i}x_{e}\left(t\right)\right) \end{array}$$

The biggest estimated power for each target frequency is considered the detected frequency (Mendoza-Montoya, 2018).

### 2.3. Experimental protocol

**Figure 2:**
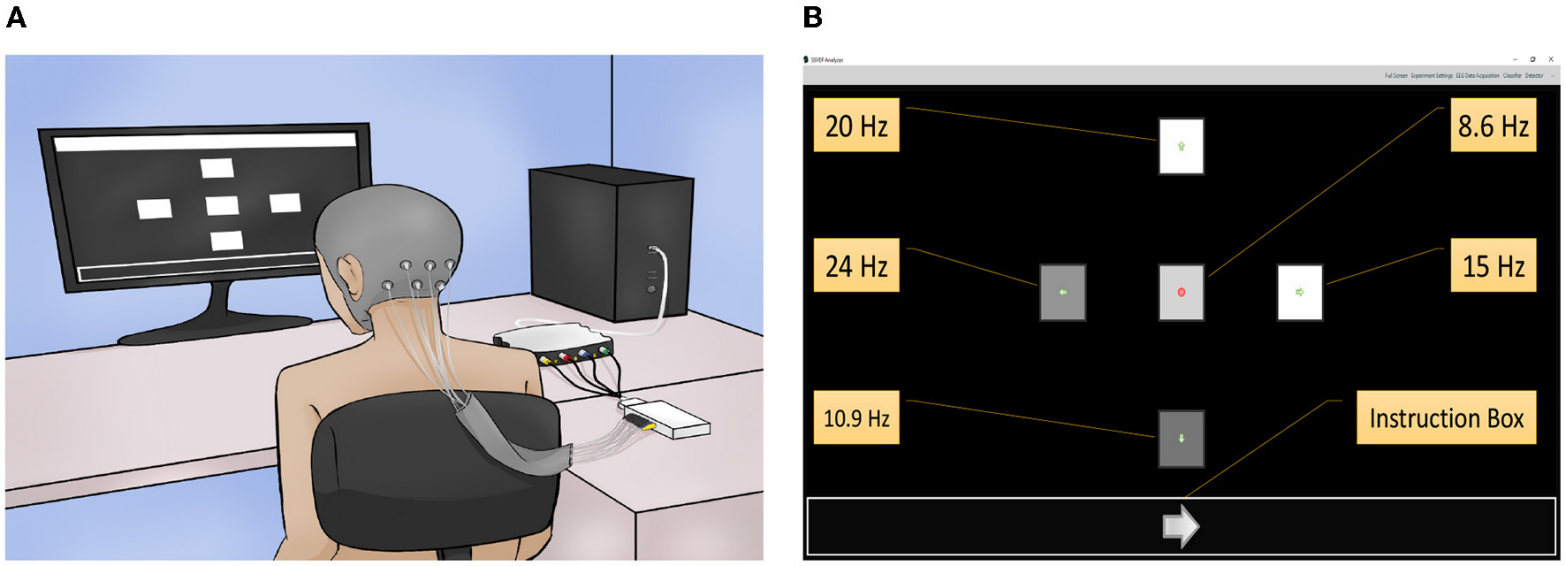
Description of the experimental protocol. **(A)** Sketch of the experimental setup with a participant, the EEG recording system, and the computer screen with the GUI displaying the five visual stimuli and the instruction box. **(B)** Configuration of the GUI with the five visual stimuli and their stimulation frequencies: 8.5714 Hz in the center, 10.9091 Hz in the bottom, 15 Hz in the right, 20 Hz in the top, and 24 Hz in the left square symbol.

The experiments were carried out in an acoustically isolated room. Participants were seated in a comfortable chair in front of a 27-inch Samsung computer screen, model C27RG50FQL, with 1920 × 1080 resolution and 240 Hz refresh rate. A Graphical User Interface (GUI) was displayed on this screen with five squares evenly distributed and with an instruction box at the bottom. The squares, with dimensions of 116 × 116 pixels, were used as a visual stimulus and therefore they flickered at a specific frequency. The instruction box, with dimensions of 1920 × 116 pixels, was used to guide the users on the execution of the experiment. Figure 2A shows a sketch of the experimental setup with a participant, the EEG recording system, and the computer screen with the GUI. Figure 2B shows the GUI with the five squares used as a visual stimulus, their flickering frequencies, and the instruction box.

The flickering of each square was performed at a specific frequency depending on the refresh rate of the monitor (240 Hz). The square located at the center, bottom, right, top, and left flickered at a frequency of 8.5714, 10.9091, 15, 20, and 24 Hz, respectively. These frequencies were selected because they are not multiples of each other, and they are in the range that has been commonly used in previous studies to induce SSVEP responses (Ng et al., 2012; Cysewska-Sobusiak and Jukiewicz, 2016; Chen et al., 2019). These studies suggest that the stimulation frequencies that generate the strongest SSVEP responses are in the range of 5 to 25 Hz. As the stimulation frequencies were generated using as reference the monitor refresh rate, they are indeed 28, 22, 16, 12, and 10 times slower than the 240 Hz, respectively. Note in Figure 2B that each square contains inside them an arrow, with dimensions of 16 × 9 pixels, pointing up, down, left, and right respectively if the square is located on the top, bottom, left, and right of the screen, while the square located on the screen center contains a traffic stop symbol, with dimensions of 16 × 16 pixels. This is because the GUI, as one of its applications, has the functionality of a navigation system (Mendoza-Montoya, 2018).

**Figure 3:**
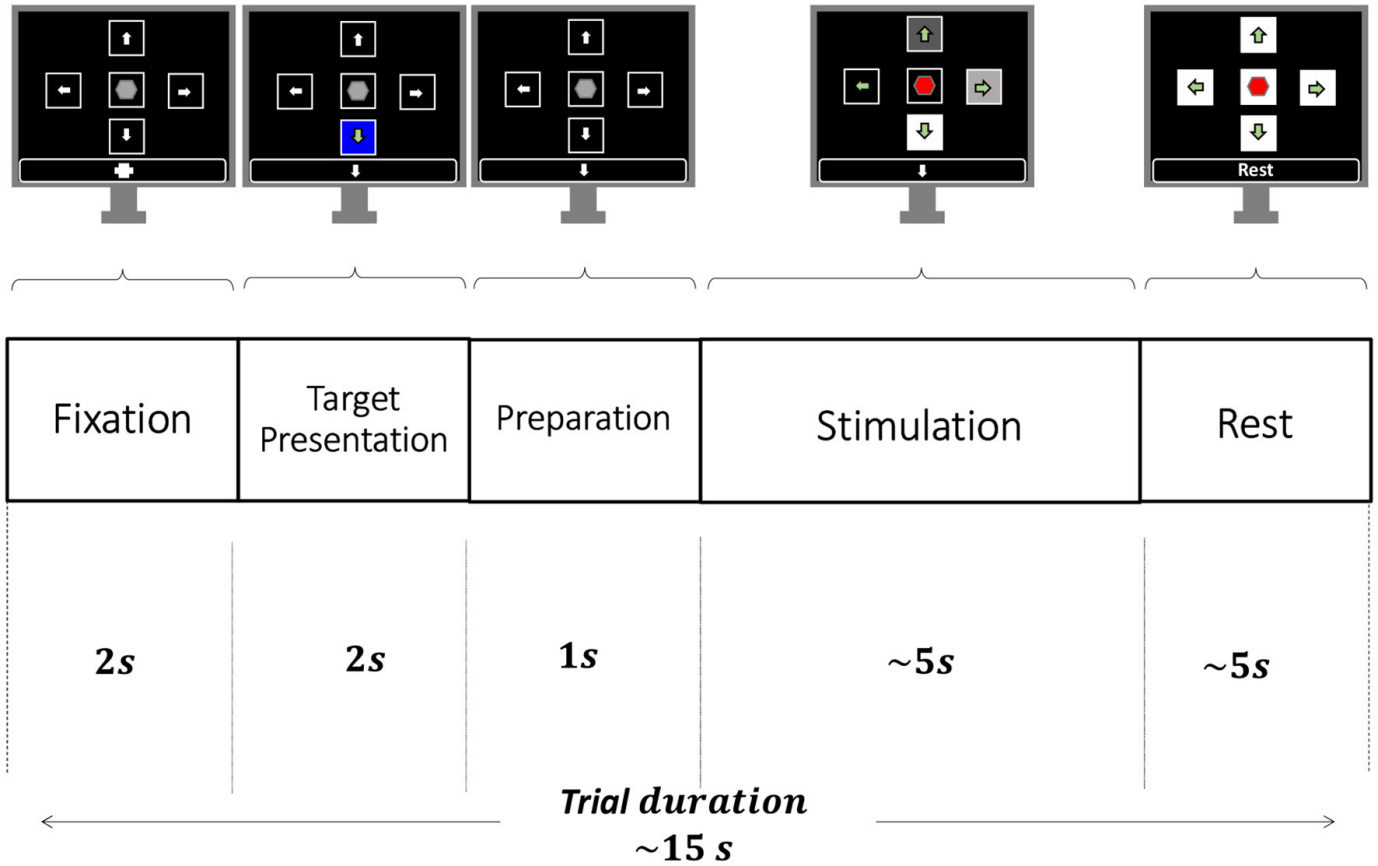
Temporal sequence of a trial. Each trial consists of five phases: *Fixation* (2 s), *Target Presentation* (2 s), *Preparation* (1 s), *Stimulation* (~5 s), and *Rest* (~5 s).

Experiments were executed in trials where the participant focused her/his visual attention on one out of the visual stimuli according to the information shown in the instruction box of the GUI. The basic timing sequence of a trial is depicted in Figure 3 and consisted of the following five phases:

1. *Fixation*. A cross symbol is shown during 2s in the information box, which indicates to be prepared and relaxed. None of the squares flickers during this phase.
2. *Target Presentation*. One of the five squares is randomly highlighted with a blue background and the corresponding arrow or stop symbol is shown in the information box. This event last 2s and indicates to the participant the specific visual stimulus that they have to focus their attention on during the subsequent *Stimulation* phase.
3. *Preparation*. None of the squares is highlighted or shown in the information box. This last 1s and indicates to be ready for the upcoming *Stimulation* phase.
4. *Stimulation*. All five squares flicker each one at its specific stimulation frequency. In this phase, participants are requested to focus their gaze on the square specified in the *Target Presentation* phase while ignoring the other visual stimuli. This visual stimulation last 5s.
5. *Rest*. None of the stimuli is highlighted and the text *Rest* is presented in the information box. This instructed the participants to rest from the experiment during 5s.

**Figure 4:**
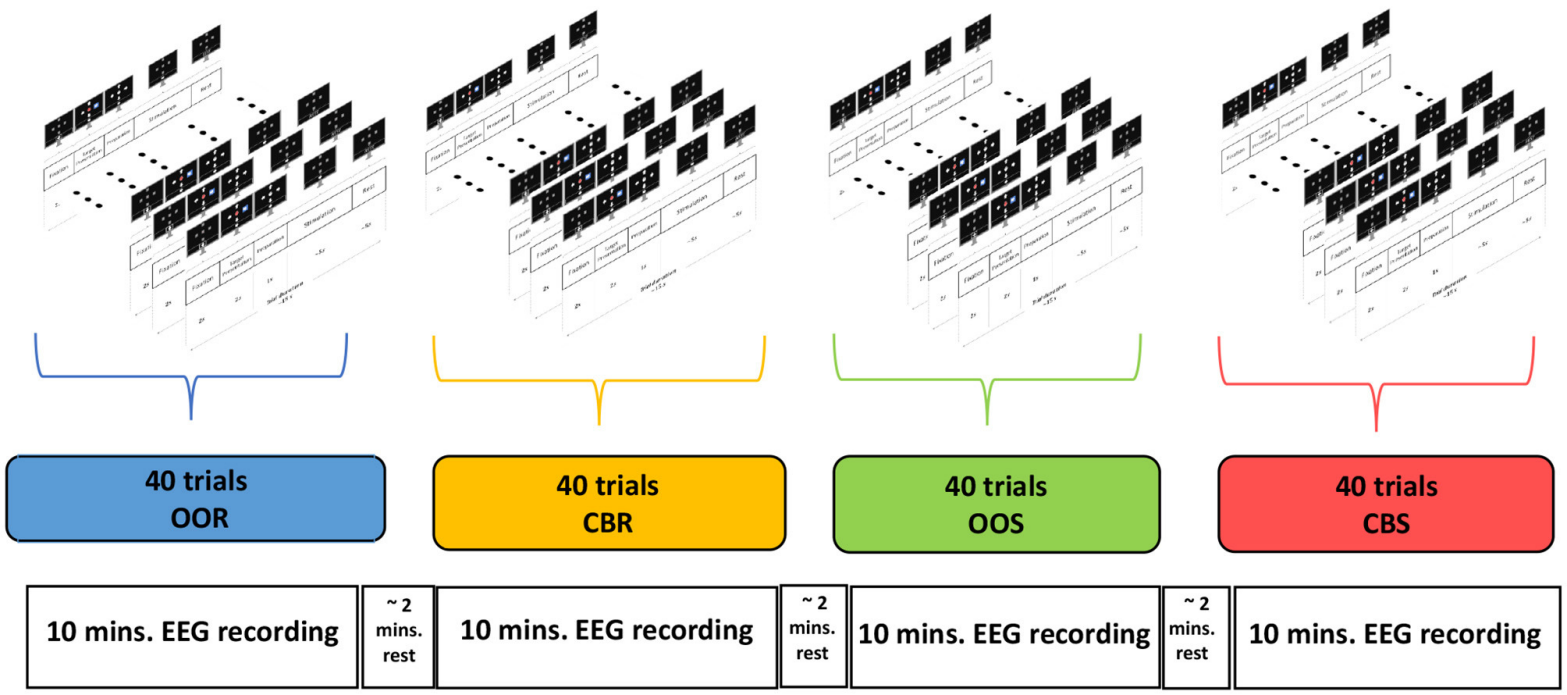
Temporal sequence of an experimental session. A session consists of four recording runs, one for each type of visual stimulus. Each run consists of 40 trials, that is, eight trials for each of the five stimulation frequencies.

For each participant, experiments were carried out in a single experimental session consisting of 4 recording runs. In each run, only one visual stimulus type (either OOR, OOS, CBR, or CBS) was employed. Moreover, the order of the visual stimulus type was randomized across runs for each of the participants. A total of forty trials were recorded per run (which represents ~10min of data recording per run), yielding eight trials for each of the five stimulation frequencies. Figure 4 shows the temporal sequence of a typical experimental session. To avoid fatigue and reduce tiredness, participants were allowed to rest between runs for about 2 or 3 min, or longer if needed. During the execution of the experiment, the participants were duly instructed to avoid moving the body or head and blinking between the *Fixation* and the *Stimulation* phases, while they were advised to move during the *Rest* phase if needed. At the end of the experimental session of a participant, four recording runs were obtained, one for each type of visual stimulation, and each run contains forty trials, that is, eight trials for each of the five stimulation frequencies.

It is important to note that during the stimulation phase, the squares are the ones that change in luminance and not the arrows or hexagon symbols located inside them. These symbols have no function other than to indicate the direction in BCI navigation applications.

### 2.4. Participants and data acquisition

Twenty-seven (27) participants (12 women and 15 men) were recruited for this study. The age range was between 18 and 24 years. All volunteers had normal or corrected vision with glasses. In the recruitment of volunteers, exclusion criteria were implemented such as having had epileptic episodes, having been diagnosed with a psychiatric disorder, or subjects with significant progressive disorders or unstable medical conditions requiring acute intervention. Participation in the study was voluntary and all subjects had the opportunity to quit the experiment at any time they wished. Each volunteer was instructed in detail about the objective of the study and the procedure to be carried out. All participants voluntarily signed an informed consent form, which complied with the standards of the Declaration of Helsinki.

To collect the EEG signals, 8 channels of a g.SCARABEO Ag/AgCl active electrodes system and a g.USBamp biosignal amplifier were used. The acquisition of EEG signals was enhanced through the inoculation of conductive gel on the active electrodes, attached to a g.GAMMAcap. The previously described instrumentation comes from the manufacturer g.tec medical engineering GmbH, Schiedlberg, Austria. According to the international 10-20 system, the channels used were *PO*7, *PO*3, *POz*, *PO*4, *PO*8, *O*1, *Oz*, and *O*2, in addition to the ground, placed to the *AFz* channel, and a reference channel, located in the right ear lobe. The EEG signals were discretized at a sampling frequency of 256 Hz. A band-pass filter in the interval between 0.5 and 60 Hz and a Notch filter configured to suppress the presence of the power line frequency were applied to the sampled recordings.

The interface used in our experiments (Mendoza-Montoya, 2018) allows registering, together with the 8 EEG channels, an additional channel containing the time markers associated with each of the five events generated from the computer during the occurrence of a trial. Thus, for each trial, there are labels indicating the precise moment when the fixation cross is presented to each participant, the target to be focused on during the subsequent stimulation stage, the preparation instant, the stimulation phase, and the resting phase. The encoding of these time marks is presented in detail in the description of the database, located at https://zenodo.org/record/7758425#.ZBvGmnbMLIW.

The user comfort was associated with the performance of the representative visual stimuli. This was subjectively measured by a two-question survey applied to each participant at the end of each experiment. The survey questions were:

- *For the four types of stimuli (OOR, OOS, CBR, CBS), was it possible to distinguish the type of modulation (rectangular from sinusoidal) on the luminous intensity of the stimulus?*
- *Which of the four types of visual stimulus best contributed to keeping vision focused on the screen?*

### 2.5. Frequency analysis

**Figure 5:**
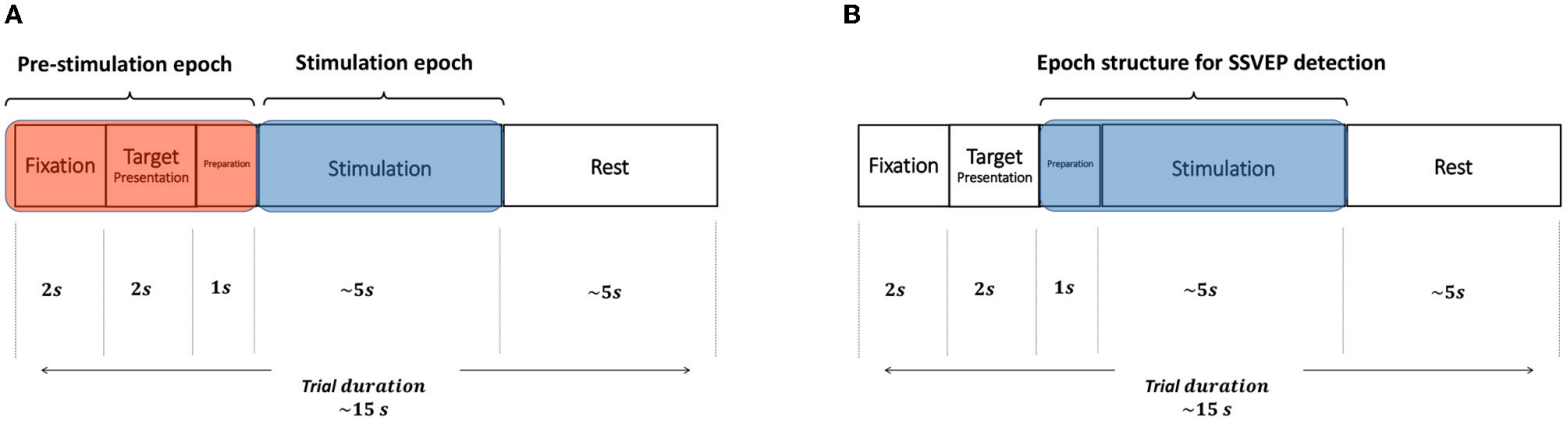
Graphical illustration of the EEG data epochs used for the frequency analysis and for the assessment of SSVEP detection. **(A)** For the frequency analysis two 5 s-long epochs were extracted from each trial, *No-stimulation* epoch (comprising the *Fixation, Target Presentation* and *Preparation* phases) and *Stimulation* epoch (containing the *Stimulation* phase). **(B)** For the assessment of SSVEP detection, one 6 s-long epoch was extracted from each trial initiating 1 s prior to the onset of the *Stimulation* phase and finishing at the end of this phase.

The goal of this analysis was to study the spectral power of the EEG signals during visual stimulation and no stimulation at all. To perform this analysis we extracted two different EEG epochs from each trial: (*i*) *Stimulation* epochs comprising EEG signals of the entire 5 s-long *Stimulation* phase; and (*ii*) *No-stimulation* epochs comprising EEG signals of the *Fixation, Target Presentation*, and *Preparation* phases, and therefore, having a duration of 5 s. Figure 5A shows how these two epochs are extracted from each trial.

This frequency analysis is essential because we expect to find larger spectral power values in *Stimulation* than in *No-stimulation* EEG response, specifically at the stimulation frequencies and its harmonics, and to determine which one of the four visual stimulation types induces the larger spectral power values during *Stimulation*.

The Power Spectral Density (PSD) method was used to compute the spectral power as this is one of the most common and robust approaches to performing frequency analysis of EEG signals (Wang et al., 2006; Nakanishi et al., 2018). The PSD was computed using the Fieldtrip toolbox (Oostenveld et al., 2010) for each of the trials corresponding to each of the five stimulation frequencies, both for the *Stimulation* and *No-stimulation* conditions. Raw EEG trials were tapered by a 2-s Hanning window (Proakis and Manolakis, 2006) with no overlapping. Then, the Fast-Fourier-Transform (FFT) of the data was taken. This was done for frequencies between 2 and 50 Hz with steps of 0.5 Hz.

The spectral power of the *Stimulation* and *No-stimulation* conditions were studied for each of the four visual stimulus types (OOR, OOS, CBR, and CBS) and each of the five stimulation frequencies (8.5714, 10.9091, 15, 20, and 24 Hz). The non-parametric paired data statistical test Wilcoxon rank-sum test was used to determine significant differences between the two conditions. Statistical analyses were carried out separately for each channel (8 in total) and frequency (96 in total), resulting in channel-frequency maps of statistical significance that allow visual inspection of the channels and frequencies where there are and there are no significant differences between the two conditions. These statistical tests were performed at a confidence level of α = 0.01. Bonferroni correction was applied to account for the multiple comparisons errors (Henry, 2015) due to the number of channels and frequencies.

Another frequency analysis implemented was the estimation of the wide-band signal-to-noise ratio (SNR), defined in Equation (13). This parameter allows us to properly characterize the broadband noise as well as the contribution of harmonics to the signals (Liu et al., 2020).


(13) $$\begin{array}{l} SNR=10log_{10}\frac{\sum_{k=1}^{k=Nh}P\left(k.f_{i}\right)}{\sum_{f=0}^{f=f_{s}/2}P\left(f\right)-\sum_{k=1}^{k=Nh}P\left(k.f_{i}\right)} \end{array}$$

Here *Nh* represents the number of harmonics (*Nh* = 4), *P*(*f*_*i*_) denotes the power spectrum at each stimulation frequency *f*_*i*_, and *f*_*s*_/2 is the Nyquist frequency.

### 2.6. Assessment of SSVEP detection

To carry out this analysis we extracted epochs comprising EEG signals from 1s before the initiation of the *Stimulation* phase and up to the end of this phase. Hence, the duration of the epochs was 6s. Epochs were time re-referenced to the initiation of the *Stimulation* phase, that is, *t* = 0 and *t* = 5s represent the initiation and the end of the visual stimulation, respectively, while there is no stimulation during −1 ≤ *t* < 0s. Figure 5B illustrates how an epoch is extracted from a trial to perform SSVEP detection.

**Figure 6:**
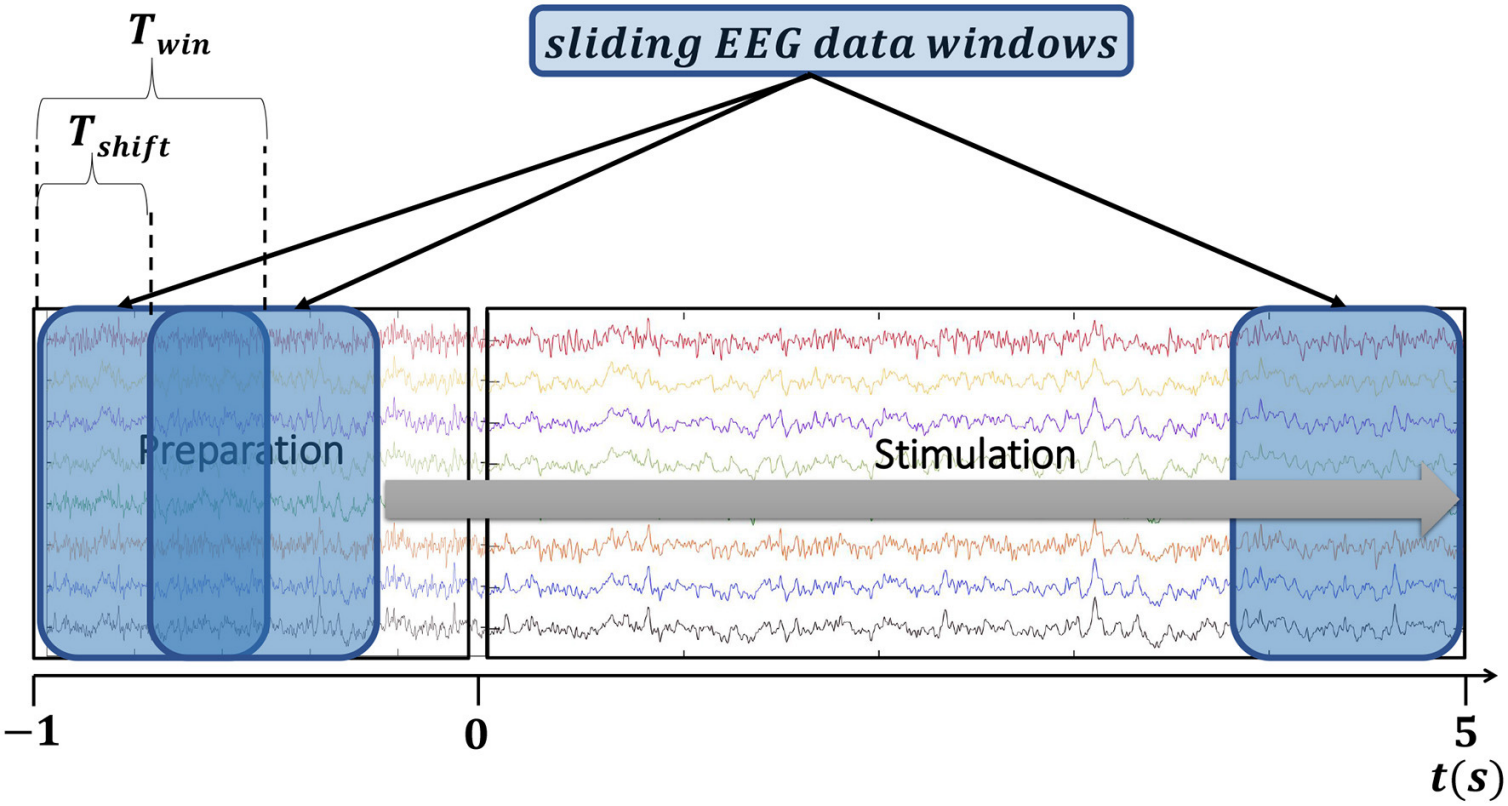
Illustration of the EEG data windows used to carry out SSVEP detection in a 6 s-long data epoch.

Given an EEG data epoch, the detection of the stimulation frequency was carried out using sliding EEG data windows of length *T*_*win*_ seconds in steps of *T*_*shift*_ seconds. Figure 6 illustrates the process employed to detect the stimulation frequency in an epoch. This procedure was chosen because it allows calculating the stimulation frequency over time as new EEG observations are available resembling the case of a realistic online situation. Following previous studies (Chen et al., 2015b; Nakanishi et al., 2018; Liang et al., 2020), in our analysis we used a time window of length 1s (*T*_*win*_ = 1*s*) and steps of 0.05s (*T*_*shift*_ = 0.05*s*). We considered other window lengths and steps and these two values provided better performance in terms of accuracy and detection time.

The detection of the SSVEP responses was assessed for each combination of detection methods (CCA, FBCCA, and MEC) with visual stimulus type (OOR, OOS, CBR, and CBS), in each one of the stimulation frequencies (8.5714, 10.9091, 15, 20, and 24 Hz). The following metrics were computed to assess performance in the detection of the SSVEP responses:

- *Detection Accuracy (DA)*. This metric measures the percentage of correct detections. For the estimation of DA, the number of correct detections *N*_*detects*_ was computed concerning the total number of trials *N*_*Trials*_ according to *DA* = *N*_*detects*_/*N*_*Trials*_. One DA value was obtained for each stimulation frequency and visual paradigm.
- *Detection Time (DT)*. This metric measures the time elapsed to decide the final stimulation frequency in an epoch as in the case of a realistic online situation of SSVEP detection. Therefore, the detection time in an epoch was computed as the time at which three consecutive windows are associated with the same stimulation frequency. We decided to use three consecutive time windows to choose the stimulation frequency of an epoch because this allows us to select it as in an online setup. Finally, the average Detection Time across trials was calculated, obtaining a value for each stimulation frequency and visual paradigm.
- *Information Transfer Rate (ITR)*. This metric estimates the online BCI performance, however, we propose to use this measure to assess the pseudo-online evaluation of SSVEP target classification, using as parameters the previously defined DA, the speed with which a target is correctly chosen (DT), and the number of targets (Volosyak, 2011). Thus, the ITR was calculated across-all epochs by the following equation:
  (14) $$\begin{array}{l} ITR=s\left[\log\left(N\right)+DA\log\left(DA\right)+\left(1-DA\right)\log\left(\frac{1-DA}{N-1}\right)\right] \end{array}$$
  where *s* = 60/*DT* is the number of commands performed per minute, *N* is the number of targets (in our case *N* = 5) and *DA* is the detection accuracy.

Statistical non-parametric Kruskal-Wallis test was applied to assess significant differences between distributions of DA, ITR, and DT respectively, for the four visual paradigms and the three detection methods and this way obtain the winning combination in each of the three performance parameters. All statistical tests were carried out at a confidence level of α = 0.05.

### 2.7. Dataset description

Each participant performed one session consisting of a 5-target SSVEP selection task, giving four data files per subject at the end of each experiment. The four data files are according to the four stimuli types: OOR, OOS, CBR, and CBS. The raw EEG signals along with a detailed description of the recorded data are freely available and can be accessed to an open-access site with https://zenodo.org/record/7758425#.ZBs5DnbMLIU. The database for this study is also available on request to the corresponding author.

## 3. Results

The results obtained from the analysis of SSVEP signals are presented below. The main objective was to study the brain response to different combinations of visual stimuli (On-Off and Checkerboard) and light pattern modulating waveforms (sinusoidal and rectangular pulse), and second, to investigate the effect on the classification accuracy of applying three SSVEP detection methods (MEC, CCA, and FBCCA) to the EEG signals obtained after presenting four types of visual paradigm. Finally, we also assessed the visual comfort of users by applying different visual paradigms that elicit an SSVEP response.

### 3.1. User perception

In the user comfort assessment, we found that out of the 27 participants, only 5 stated that they were able to distinguish the rectangular signal modulation from sinusoidal signal modulation, for both, On-Off and checkerboard patterns, thus compiling an 18.5%. Out of the 27 subjects, 51.9% expressed a predilection for the checkerboard pattern in terms of comfort and focus, regardless of the modulating signal shape.

### 3.2. Frequency analysis

In the results, we initially compare the power response corresponding to the *Stimulation* vs. *No-Stimulation* conditions for each of the eight channels and the four visual paradigms. Subsequently, we compared the power of the brain responses for each of the four visual paradigms applied during the *Stimulation* stage. This was done for all participants and the five stimulation frequencies generated in the GUI. A frequency-domain analysis of the signals was performed to evaluate which of the four visual paradigms elicited the strongest SSVEP response at each of the 5 stimulation frequencies. For this purpose, the PSD was obtained by calculating the FFT of each trial to finally obtain the average power characteristic for each stimulation frequency and each visual paradigm. We found that the spectral power at the stimulation frequency is higher in the *Stimulation* phase than in the *No-Stimulation* phase. We also obtained greater power in the brain response to visual paradigms with On-Off stimulation, regardless of the modulating waveform of the visual stimulus, for each of the five stimulation frequencies.

**Figure 7:**
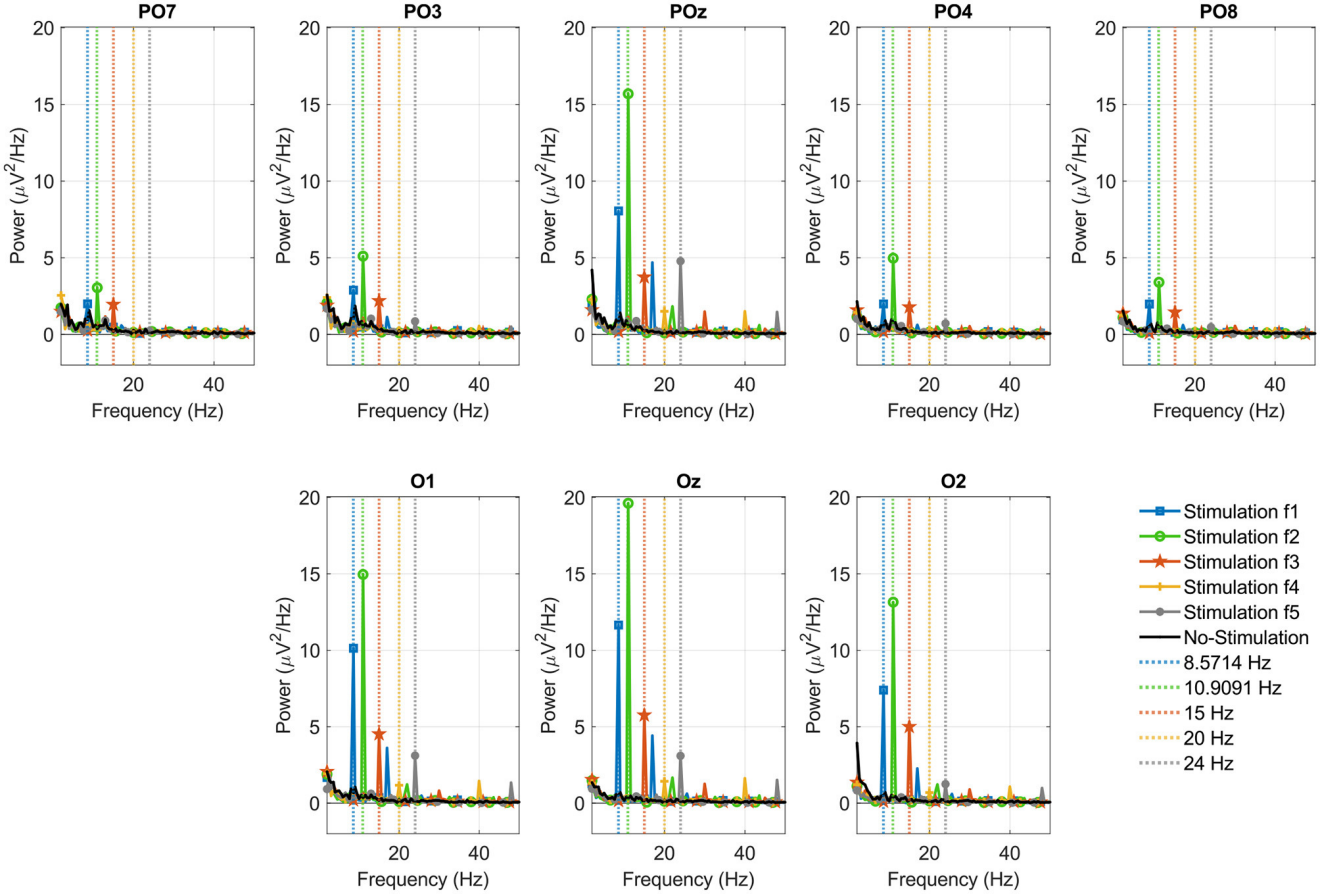
EEG average power response per channel of one participant (subject 27). For each stimulation frequency, the SSVEP responses are presented in colors corresponding to the Stimulation (blue, green, red, yellow, and gray) and No-Stimulation (black) stages when the visual OOR paradigm is applied at the following frequencies: 8.57 Hz (dashed blue vertical line), 10.909 Hz (dashed green vertical line), 15 Hz (dashed red vertical line), 20 Hz (dashed yellow vertical line), and 24 Hz (dashed gray vertical line).

To illustrate each of these results, Figure 7 shows the EEG average power response for the five stimulation frequencies when Participant 27 was subjected to the OOR paradigm. Each of the five stimulation frequencies is represented by a vertical dashed line of a specific color. Thus, the application of a stimulus at a frequency of 8.57 Hz is represented in blue, the stimulus at a frequency of 10.909 Hz is represented in green, the stimulus at a frequency of 15 Hz is represented in red, the stimulus at a frequency of 20 Hz is represented in yellow and finally, the stimulus at a frequency of 24 Hz is represented in gray. The brain response to the application of the visual OOR paradigm during the *Stimulation* stage is represented with the color previously described corresponding to each stimulation frequency. The brain response during the *No-Stimulation* stage is represented in black. Additionally, we incorporated in the Supplementary material document the comparison plots of the EEG average power during the Stimulation vs. No-Stimulation stages for the five stimulation frequencies applied to one participant (subject 27). In each of the eight channels, the highest power peaks match the stimulation frequency represented by a vertical dashed line. Except for the *PO*_*z*_ electrode, a difference in amplitude of the peak values in the occipital channels with respect to the parietal-occipital channels can also be distinguished. The response of the central channels (*PO*_*z*_ and *O*_*z*_) for this participant, as in most of the subjects, is superior to that of the other channels.

Table 1 shows the average power values across all channels and subjects and each visual paradigm. For each stimuli frequency (column) the maximum power values have been shaded for each of the four visual paradigms applied. These results are across all participants during the experimental session. For stimulation frequencies 1 and 4 (8.57 and 20 Hz) a maximum average value was obtained when applying the OOR paradigm, while for stimulation frequencies 2, 3, and 5 (10.909, 15, and 24 Hz) the maximum average power values were obtained when applying the OOS paradigm. In the column representing the average power across all stimulation frequencies, the maximum value corresponding to the OOS visual paradigm is highlighted in gray.

**Table 1:** Average power (in μ*V*^2^/*Hz* units) across-all participants, trials, and channels for each type of visual stimulus at the five target frequencies (*f1* = 8.57 Hz, *f2* = 10.909 Hz, *f3* = 15 Hz, *f4* = 20 Hz, and *f5* = 24 Hz).

**Stimulus type**	***f1***	***f2***	***f3***	***f4***	***f5***	**Across-stimulation frequencies Power average**
OOR	**1.48 ± 2.53**	1.85 ± 2.51	1.64 ± 1.55	**1.02 ± 1.17**	0.75 ± 0.69	1.35 ± 1.69
OOS	1.32 ± 1.59	**2.02 ± 2.88**	**1.64 ± 1.81**	1.02 ± 1.33	**0.92 ± 1.11**	**1.38 ± 1.74**
CBR	1.00 ± 1.20	0.29 ± 0.29	0.33 ± 0.46	0.37 ± 0.41	0.30 ± 0.31	0.46 ± 0.53
CBS	1.01 ± 1.32	0.33 ± 0.41	0.34 ± 0.42	0.36 ± 0.48	0.32 ± 0.39	0.47 ± 0.60

The values per stimulation frequency (columns) that yielded the highest power values for each type of stimulus at each target frequency are highlighted (gray-highlighted values).

The results of the wide-band SNR estimation are reported in Table 2. SNR values are presented across participants, trials, and channels for each of the four visual paradigms and the five stimulation frequencies. The OOS visual paradigm exhibits higher values than those of the other paradigms for the five stimulation frequencies. To better distinguish in the table, the results corresponding to this type of visual stimulus have been highlighted with a gray background. On the other hand, the lowest values are concentrated in the CBR paradigm, except for the value corresponding to the stimulation frequency of 10,909 Hz (−13.20 dB), associated with the CBS visual paradigm. Another aspect to note is the decrease in SNR as the stimulation frequency increases for the four visual paradigms.

**Table 2:** Across subjects, trials, and channels wide-band SNR (in dB units) for each type of visual stimulus and stimulation frequencies, considering Nh = 4, where Nh is the number of harmonics.

	***f1***	***f2***	***f3***	***f4***	***f5***	**Across-stimulation frequencies SNR**
OOR	−9.80	−9.60	−10.67	−12.79	−13.80	−11.33
OOS	**−9.79**	**−9.36**	**−10.47**	**−12.46**	**−13.08**	**−11.03**
CBR	−11.06	−12.88	−15.59	−16.47	−17.38	−14.68
CBS	−10.74	−13.20	−15.48	−16.12	−17.15	−14.54

The values per stimulation frequency (columns) that yielded the highest SNR values for each type of stimulus are highlighted (gray-highlighted values).

### 3.3. SSVEP detection methods

**Figure 8:**
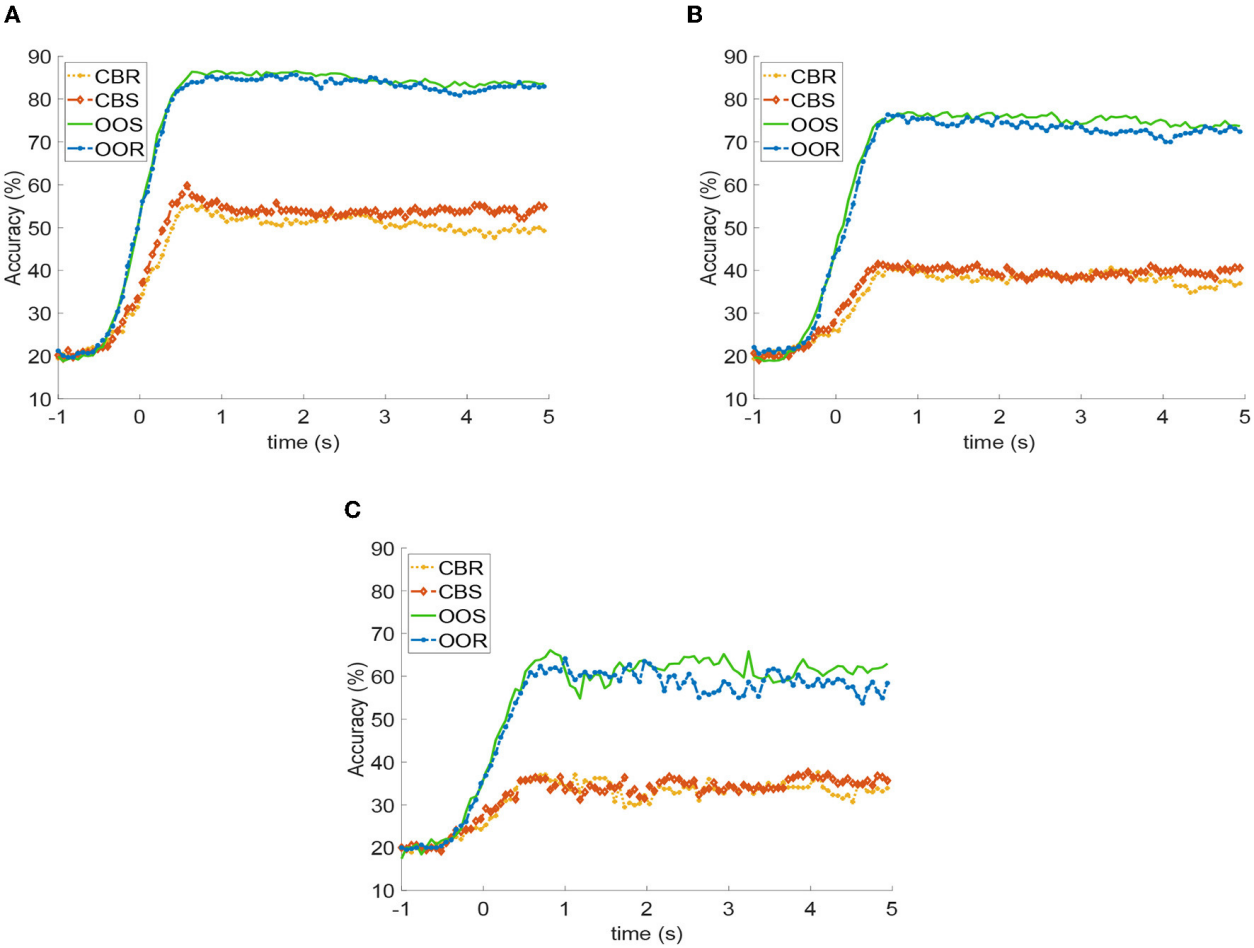
Detection Accuracy (DA) curves vs. time for each visual paradigm across subjects, trials, channels, and stimulation frequency: **(A)** FBCCA, **(B)** CCA, **(C)** MEC.

Figure 8 illustrates the detection accuracy as a function of time across participants, channels, trials, and stimulation frequencies. This was obtained for each of the four visual paradigms and the three detection methods. For the three detection methods, there is a marked difference between stimulation with OOR and OOS paradigms concerning CBR and CBS, the former being superior. Peak performance in all four visual paradigms is achieved after the first second of visual stimulus application. Additional time-dependent performances for each stimulation frequency for the three detection methods are reported in Supplementary Figure 7.

**Figure 9:**
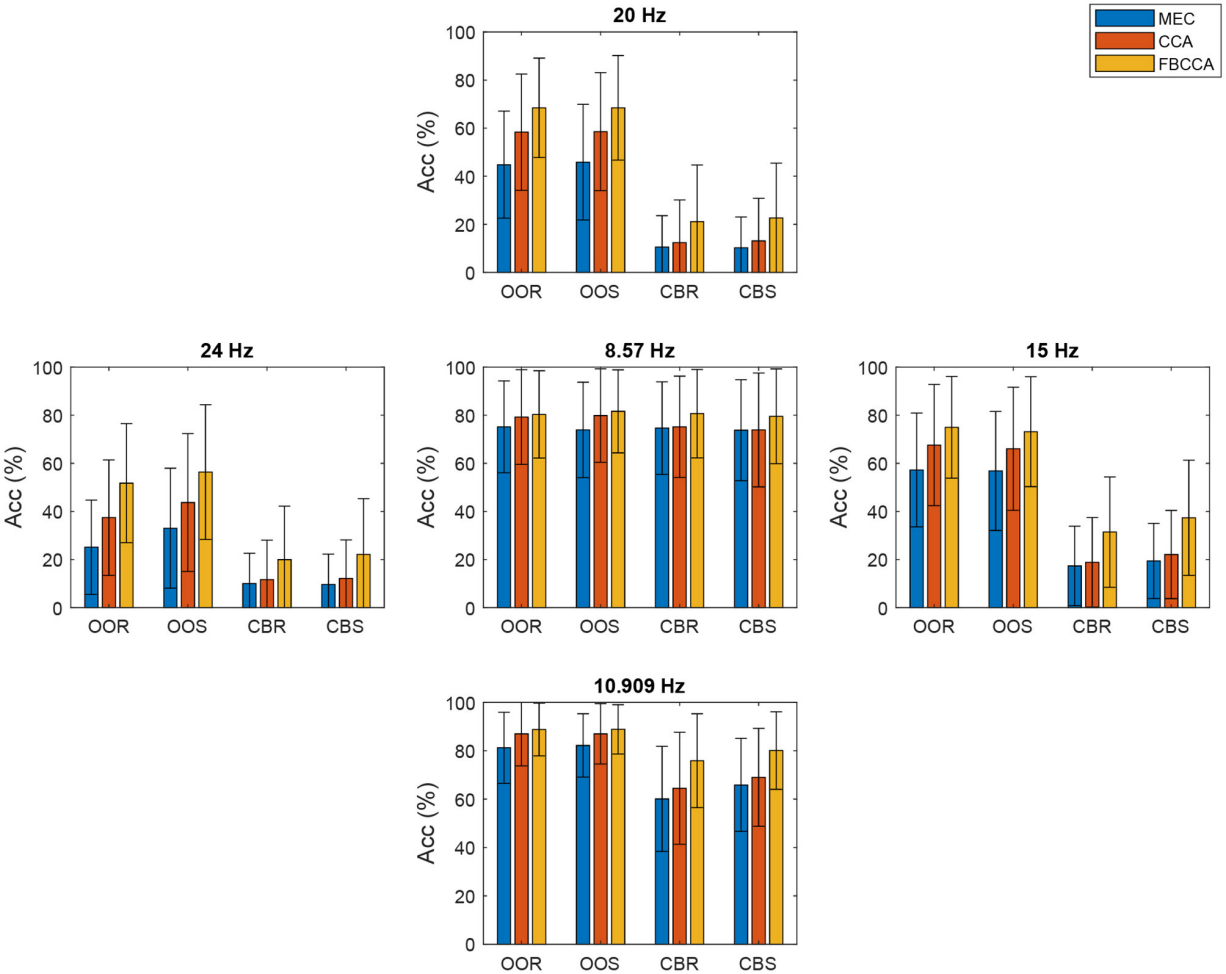
Across-all participants average accuracy for each stimulation frequency, stimulation pattern, and SSVEP detection method.

Figure 9 depicts all participant's representative bar graphs of the average accuracy when classifying target vs. non-target events with its corresponding standard deviation at each stimulus frequency for each visual paradigm and with each SSVEP detection method applied. The FBCCA detection method combined with the OOR or OOS visual paradigms outperforms the alternatives involving the checkerboard paradigm in the five stimulation frequencies presented to the users. Also, the MEC detection method combined with the CBR stimulus type yields the worst results in performance at each of the five frequencies applied. The maximum average accuracy (88.83%) was obtained with the FBCCA method for the frequency of 10.909 Hz with the OOS paradigm. The minimum average accuracy value (9.60%) was obtained with the MEC method for the frequency of 24 Hz applying the CBS visual paradigm. This accuracy rate is below the chance level.

**Figure 10:**
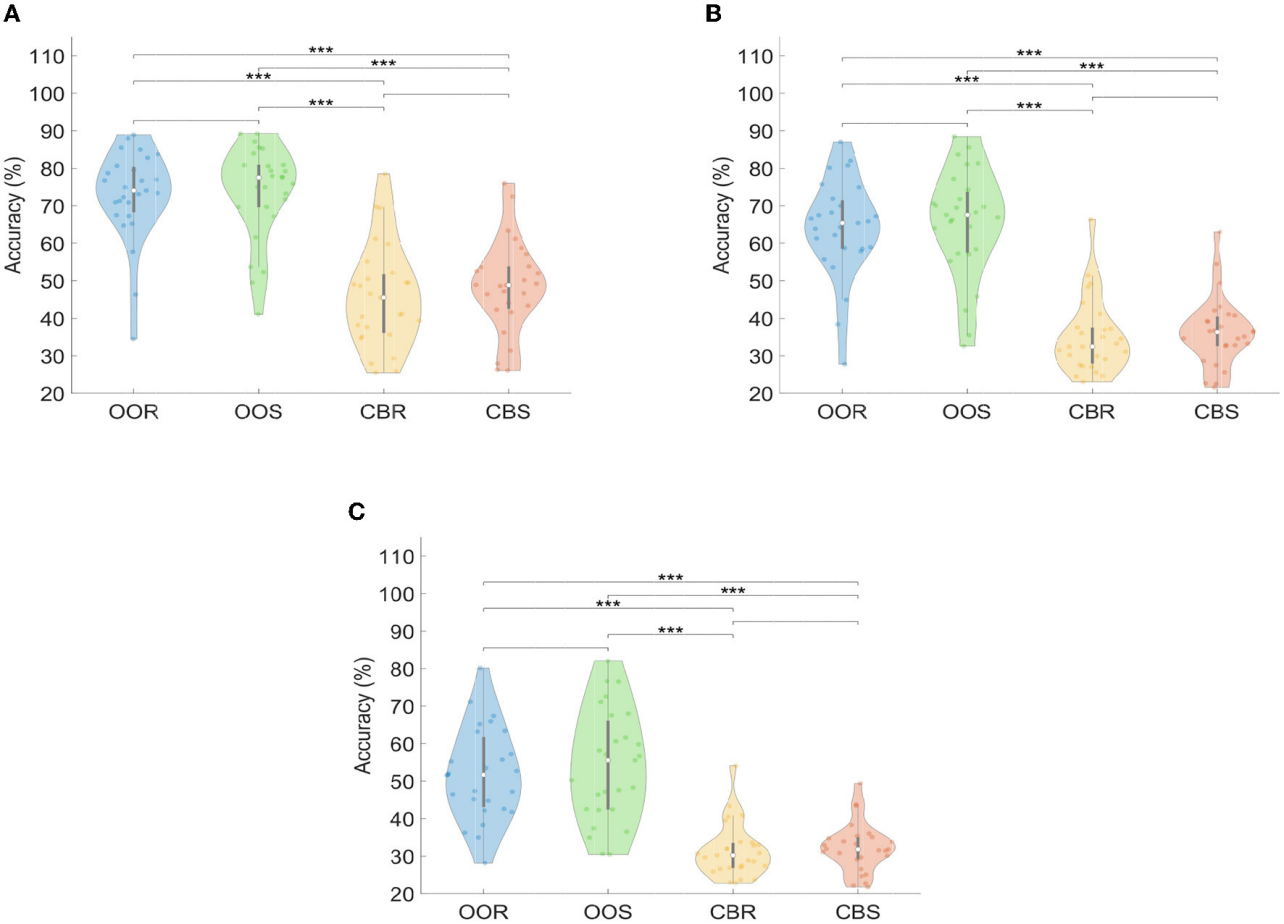
Violin-plot representation of Detection Accuracy (DA) curves for each visual paradigm across trials, channels, and stimulation frequency: **(A)** FBCCA, **(B)** CCA, **(C)** MEC. Significant differences between pairs of groups of visual paradigms are represented with asterisks, such that ***p ≤ 0.001; no asterisk above the bracket means “not significant”.

Figure 10 shows the violin plot representation of the distribution of DA values for each visual paradigm (OOR, OOS, CBR, and CBS) across trials, channels, and stimulation frequencies, and for each of the three SSVEP detection methods (FBCCA, CCA, and MEC). Violin plots allow us to appreciate the nature of multi-modal distributions of numerical data, providing information about its dispersion or concentration. Each plot shows the probability density of the DA values, with the thicker parts representing regions with more data points. The violin plots show that the medians corresponding to the OOR and OOS distributions are larger than the CBR and CBS distributions. Particularly, the median corresponding to the distribution of the OOS visual paradigm is the highest in the three SSVEP detection methods. There is a greater dispersion of the data in the CBR and CBS distributions for the FBCCA method and in the OOR and OOS distributions for the MEC method, while for the CCA method, the distributions corresponding to the four visual paradigms are more concentrated around their medians. The results of the Kruskal-Wallis and multiple comparison test indicated that there were significant differences in DA across the 12 experimental conditions (four visual stimuli paradigms by three no-training detection methods) for the FBCCA detection method (Figure 10A). Specifically, there were significant differences in DA between OOR and CBR; OOR and CBS; OOS and CBR, and OOS and CBS (*p* ≤ 0.001). However, there were no significant differences in DA between OOR and OOS and CBR and CBS. It is important to note that this pattern of results was consistent across all three detection methods (FBCCA, CCA, and MEC). These results suggest that the choice of visual stimuli paradigm can significantly impact the accuracy of SSVEP detection and that OOR and OOS paradigms may be more effective than CBS and CBR independent of the specific detection method being used.

Table 3 shows the average detection time values for each stimulation frequency and visual paradigm. The minimum detection time (0.47 ± 0.40*s*) was obtained for the frequency of 8.57*Hz* with the CBR paradigm in the FBCCA method, while the maximum value (1.51 ± 1.45*s*) was found for the frequency of 20*Hz* for the CBR paradigm in the MEC method. Considering the five stimulation frequencies, three of them (15, 20, and 24 Hz) show the shortest detection time values when the CCA method was applied with the OOR and CBS paradigms. The detection times obtained with the MEC method yielded the slowest response values for the five stimulation frequencies and the four visual paradigms when compared to the CCA and FBCCA methods. Finally, the column representing the detection time across all stimulation frequencies shows that the lowest value corresponds to the visual OOS paradigm for the CCA detection method with a value of 0.93 ± 1.08 s. This cell is highlighted in the gray background for better distinction.

**Table 3:** Across-all participants and trials average detection time (in s units) for each type of visual stimulus at the five target frequencies (*f*1 = 8.57 Hz, *f*2 = 10.909 Hz, *f*3 = 15 Hz, *f*4 = 20 Hz, and *f*5 = 24 Hz).

**SSVEP detection method**	**Stimulus type**	***f1***	***f2***	***f3***	***f4***	***f5***	**Across-stimulation frequencies Detection time**
*FBCCA*	OOR	0.90 ± 1.01	1.10 ± 1.07	0.97 ± 0.92	0.98 ± 1.00	1.09 ± 1.11	1.01 ± 1.02
	OOS	1.00 ± 0.95	0.99 ± 1.06	1.00 ± 1.04	1.02 ± 1.01	1.02 ± 1.07	1.01 ± 1.03
	CBR	0.47 ± 0.40	0.50 ± 0.44	1.28 ± 0.96	1.49 ± 1.26	1.29 ± 1.20	1.01 ± 0.85
	CBS	1.04 ± 0.99	0.93 ± 0.94	0.96 ± 0.97	1.09 ± 1.16	1.00 ± 1.05	1.00 ± 1.02
	OOR	0.79 ± 0.91	1.02 ± 1.10	0.86 ± 1.02	0.93 ± 1.15	1.08 ± 1.20	0.94 ± 1.08
*CCA*	OOS	1.00 ± 1.08	0.84 ± 1.06	0.89 ± 1.05	1.02 ± 1.13	0.92 ± 1.10	0.93 ± 1.08
	CBR	0.52 ± 0.49	0.54 ± 0.53	1.31 ± 1.18	1.11 ± 1.31	1.20 ± 1.30	0.94 ± 0.96
	CBS	1.00 ± 1.11	0.87 ± 1.05	0.97 ± 1.11	0.92 ± 1.11	0.92 ± 1.03	0.94 ± 1.08
	OOR	0.92 ± 1.04	1.14 ± 1.18	1.01 ± 1.08	1.08 ± 1.24	1.26 ± 1.35	1.08 ± 1.32
*MEC*	OOS	1.07 ± 1.13	1.05 ± 1.18	1.00 ± 1.18	1.16 ± 1.22	1.14 ± 1.22	1.08 ± 1.19
	CBR	0.49 ± 0.46	0.51 ± 0.45	1.46 ± 1.20	1.51 ± 1.45	1.45 ± 1.40	1.08 ± 0.99
	CBS	1.13 ± 1.19	0.96 ± 1.06	1.20 ± 1.25	1.12 ± 1.23	1.00 ± 1.17	1.08 ± 1.18

The values per stimulation frequency (columns) that yielded the lowest detection time values are highlighted (gray-highlighted values).

**Figure 11:**
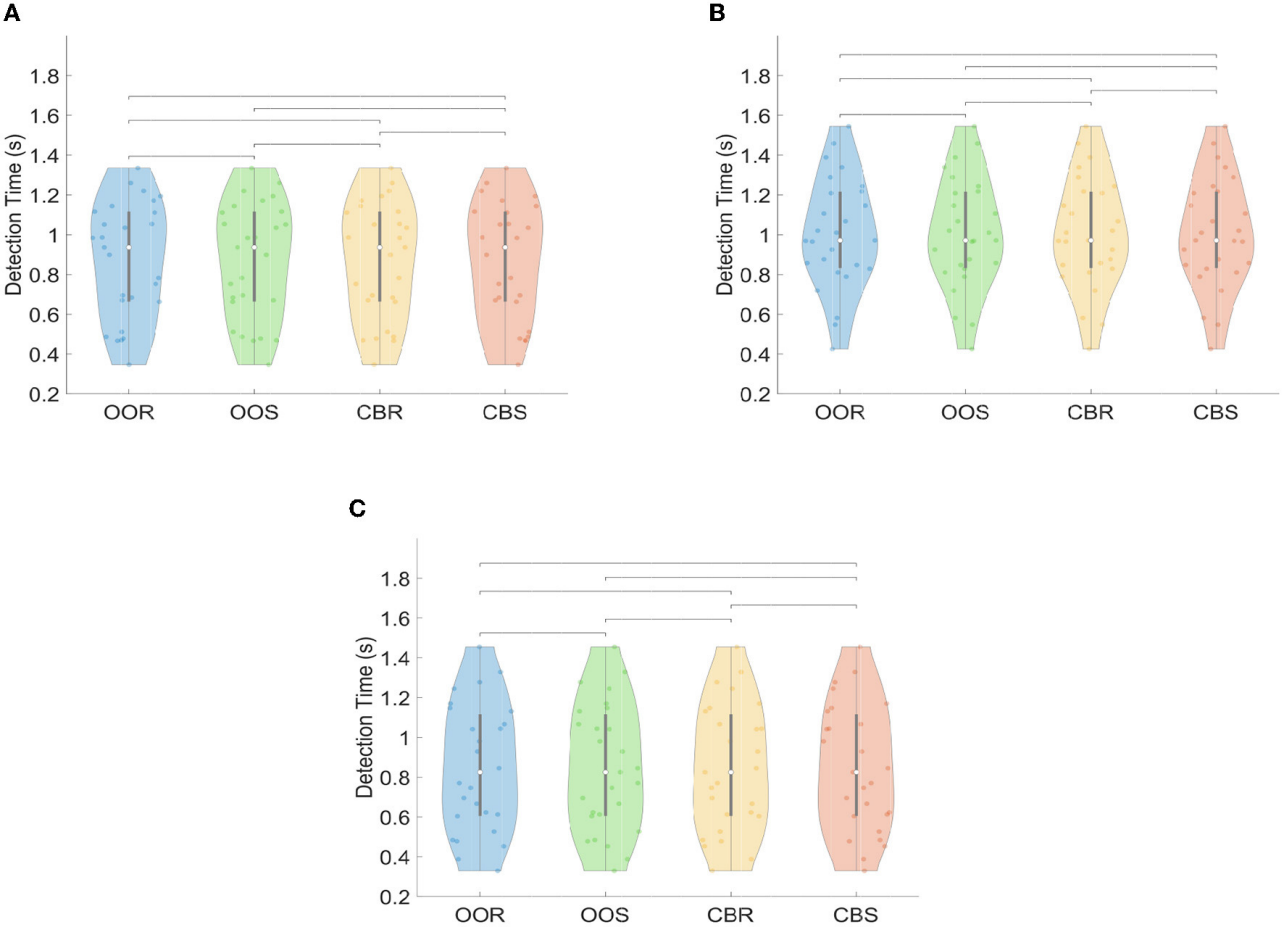
Violin-plot representation of Detection Time (DT) curves for each visual paradigm across trials, channels, and stimulation frequency: **(A)** FBCCA, **(B)** CCA, **(C)** MEC. Significant differences between pairs of groups of visual paradigms are represented such that no asterisk above the bracket means “not significant.”

The violin plots in Figure 11 illustrate the distributions corresponding to the detection time (DT) parameter for the four visual paradigms and the three SSVEP detection methods. Each graph shows that the medians of each distribution are similar and that the data distributions are well-concentrated around them. This is true for all four visual paradigms and all three detection methods. The results of the Kruskal-Wallis and multiple comparison tests indicated that there were no significant differences in detection time (DT) across the twelve experimental conditions (four visual stimuli paradigms by three no-training detection methods) for the FBCCA detection method (*p*>0.05). This pattern of results was consistent also for CCA and MEC detection methods.

**Figure 12:**
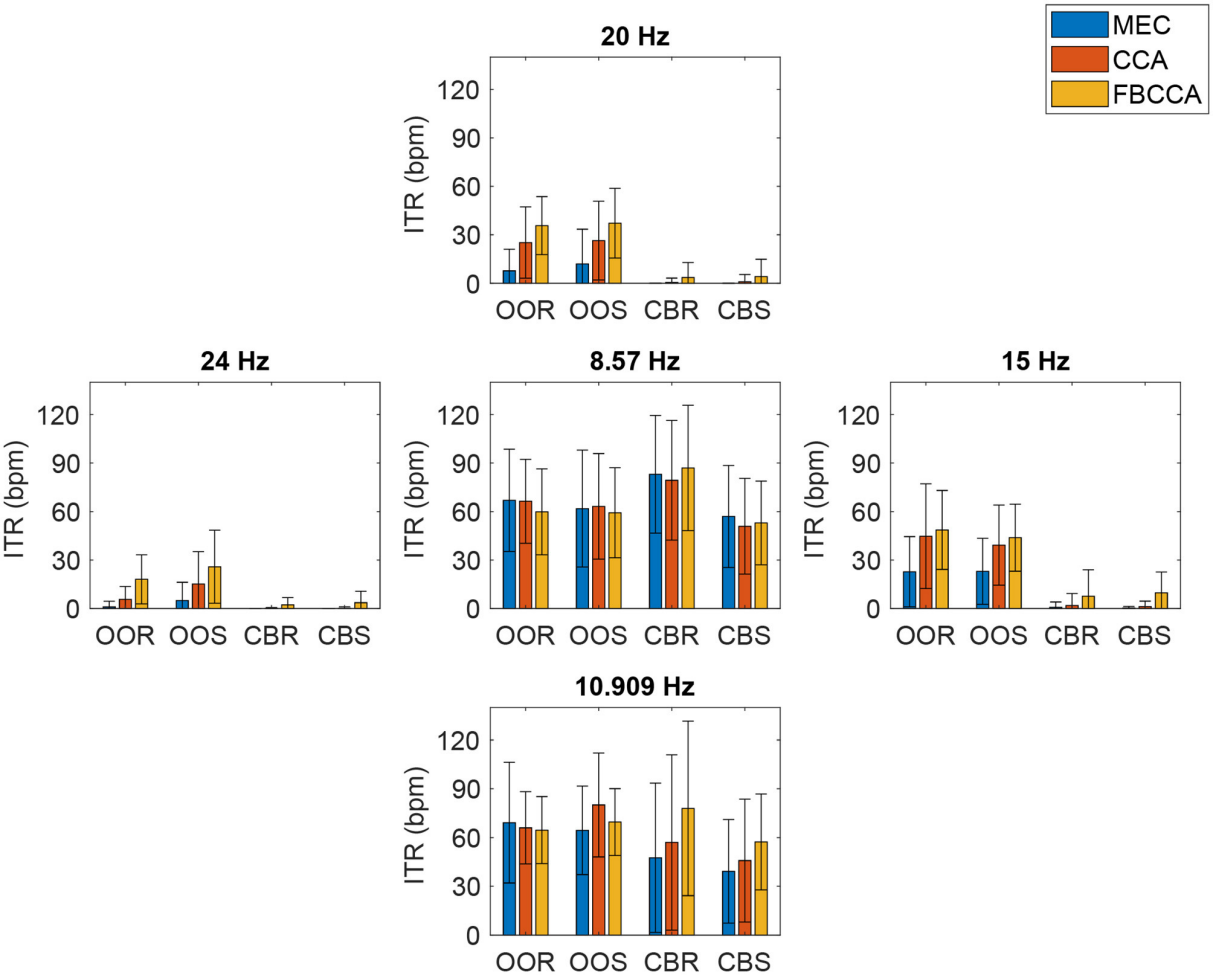
Across all participants average ITR for each stimulation frequency, visual paradigm, and SSVEP detection method.

Finally, the across subjects, channels, and trial results of the ITR, with its corresponding standard deviation, are presented (Figure 12) considering the three detection methods, the four visual paradigms, and the five stimulation frequencies. This parameter has a non-linear proportional dependence on the previously reported accuracy and is inversely proportional with respect to the detection time. As in the accuracy results, the FBCCA outperforms the other SSVEP detection methods, however, it is the CBR visual paradigm for a frequency of 8.57*Hz* that provides the maximum overall ITR value (86.95 bpm), matching the shortest average detection time (Table 3). For the CBR and CBS visual paradigms at 20 and 24 Hz frequencies, null ITR values were obtained with the CCA and MEC methods because the accuracy values were below the chance level (20%). In the first two stimulation frequencies (8.57 and 10.909 Hz) the ITR is higher for the CBR and CBS paradigms because the detection time is shorter for these cases while the accuracy values are approximately the same.

**Figure 13:**
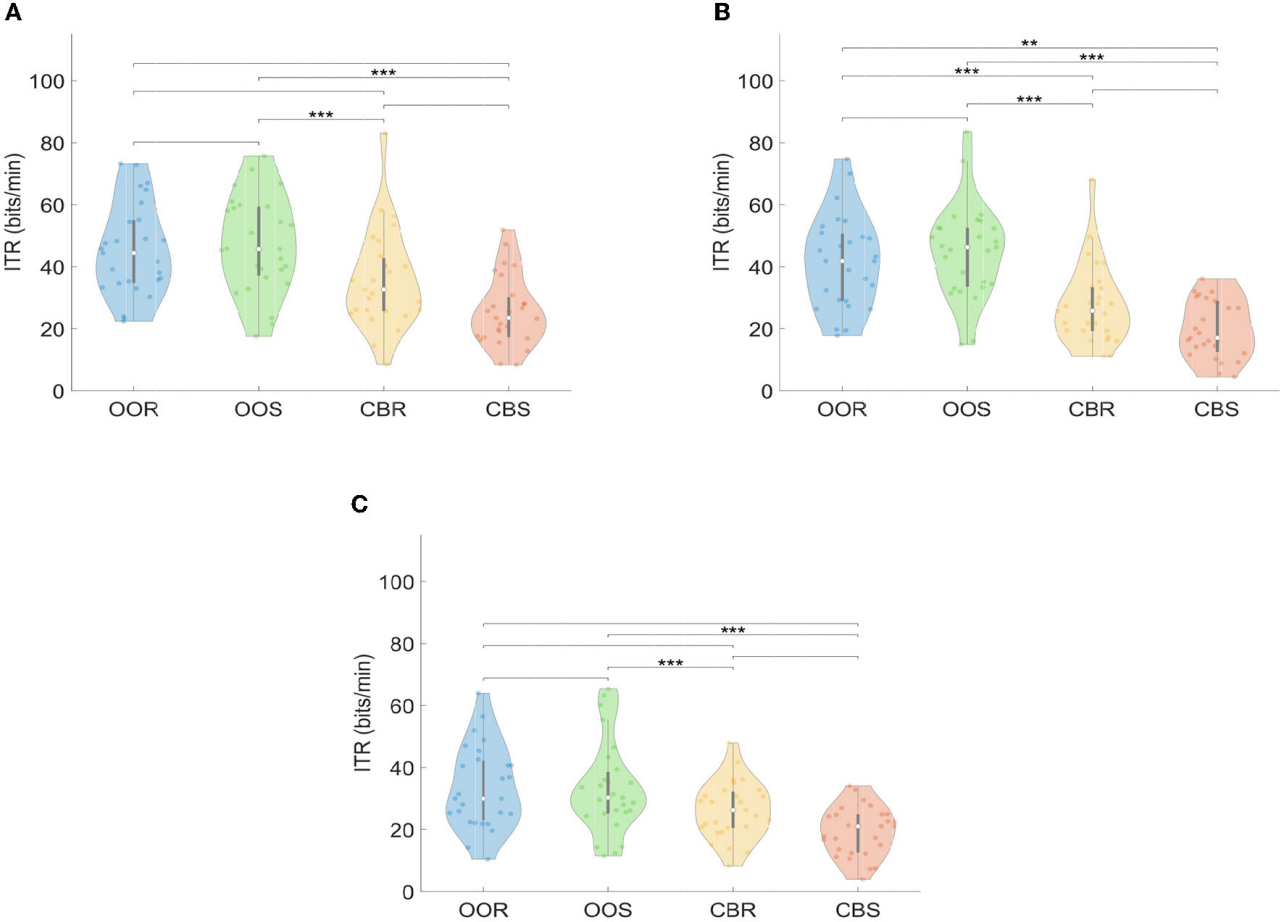
Violin-plot representation of Information Transfer Rate (ITR) curves for each visual paradigm across trials, channels, and stimulation frequency: **(A)** FBCCA, **(B)** CCA, **(C)** MEC. Significant differences between pairs of groups of visual paradigms are represented with asterisks, such that ***p ≤ 0.001; **p ≤ 0.01; no asterisk above the bracket means “not significant”.

Figure 13 shows the violin plot representation of the distribution of ITR values for each visual paradigm (OOR, OOS, CBR, and CBS) across trials, channels, and stimulation frequencies, and for each of the three SSVEP detection methods (FBCCA, CCA, and MEC). Each plot shows the probability density of the ITR values, with the thicker parts representing regions with more data points. The violin plots show that the median corresponding to the distribution of the OOS visual paradigm is the highest in the three SSVEP detection methods, the same as for the DA parameter. The results of the Kruskal-Wallis and multiple comparison tests indicated that there were significant differences in ITR in ten out of the twelve experimental conditions. In the FBCCA and MEC methods, significant differences were identified between the OOS paradigm and the two visual stimulus variants involving the checkerboard pattern (CBR and CBS) respectively. However, unlike the behavior in estimating detection accuracy, this performance did not occur in the case of the OOR paradigm vs. CBR and CBS. In the case of the CCA method (Figure 13B), of the six possible combination options, no significant differences were observed between the medians of distributions belonging to the same visual pattern, i.e., OOR vs. OOS and CBR vs. CBS, but differences were observed between OOR vs. CBS, with a significance level of 0.01, between OOR vs. CBR, OOS vs. CBR and between OOS vs. CBS, the latter three comparisons with a significance level of 0.001.

## 4. Discussion

The influence of different aspects of the visual stimulus (e.g., number of targets, stimuli frequency, the dimensions of the stimuli, stimuli colors, type of signal controlling the stimuli, number of targets, among others) on the SSVEP response and thus on BCI performance was previously reported. However, as discussed in Li et al. (2021), it is not only these parameters that influence performance but also the visual paradigm employed. It is for this reason that the objective we pursued with this work was to evaluate the performance in discriminating target from non-target elements taking into account four visual paradigms (CBR, CBS, OOS, and OOR) in conjunction with three SSVEP detection methods (CCA, FBCCA, and MEC) since, as stated in the same study, the results are inconsistent when the same SSVEP classification algorithms are applied but with different visual paradigms, demonstrating the importance of the latter in the satisfactory performance of the interface.

According to our findings, brain responses to visual stimuli showed higher average power peaks in the intervals comprising the first three stimulus frequencies (8.57, 10.909, and 15*Hz*). A similar range of stimulation frequencies has already been successfully used in other studies such as those conducted by Chen et al. (2015a) and Liu et al. (2020). To ensure a high number of targets in such a narrow range of stimulation frequencies, oscillatory stimuli consisting of combinations of frequencies and phase shifts could be incorporated, as proposed in Chen et al. (2015a) and Liu et al. (2020). The strongest brain response to visual stimuli is achieved when applying On-Off patterns and modulation of the visual stimulus is performed with a sinusoidal signal or with a rectangular pulse. The OOR and OOS visual paradigms were superior to CBR and CBS in power response in each of the channels where the signals were recorded, which is consistent with the results reported in Zerafa et al. (2013), although in this study, the type of stimulus modulation was not specified and only a flashing element was presented on the screen. It should be emphasized that the maximum average power peaks in the CBR and CBS paradigms were obtained for frequency values corresponding to twice the stimulation frequency *f*, where *f*∈ℝ; *f*:{8.57, 10.909, 15, 20, 24}*Hz*, which is consistent with the effect produced by the application of the checkerboard pattern. Our results partially disagree with those obtained in Teng et al. (2011) and Chen et al. (2019), in which the modulation of a pulsed signal with 50% duty cycle is superior to the modulation performed by a sinusoidal signal, although it should be highlighted that in the former publication the accuracy values associated with the application of a modulating signal type and not the power values, were reported. According to our findings, the dominance of one paradigm over another depends on the stimulation frequency and the stimulus type. The results also showed us that an association between the electrical brain response and the type of stimulus can be established, with a high level of confidence, but not with respect to the luminance modulating signal, since a pairwise comparison showed that there are differences between the OOR vs. CBR and CBS paradigms, that there are also differences between OOS vs. CBR and CBS, but no differences were found between the OOR paradigm with respect to OOS and CBS with respect to CBR. At the same time, this result partially matches with Cysewska-Sobusiak and Jukiewicz (2016), in which the application of the sinusoidal signal elicits the most intense brain reaction. However, three aspects should be noted here: first, the population sample reported in that study was 8 participants; second, only 2 electrodes of the international 10-20 system (*O*_1_ and *O*_2_) were used; and third, the quantitative parameter used to characterize the brain response according to the waveform of the stimulus modulating signal was the Signal-to-Noise Ratio (SNR).

In summary, our results suggest that, in the aspect related to the visual paradigms, brain responses are more prominent when subjects are exposed to an On-Off stimulation type combined with luminance modulation either by sinusoidal or rectangular signals. The results of the statistical test suggest that, in the visual paradigm, the visual pattern (pattern reversal checkerboard, on-off) is the determinant and not the luminance-modulating waveform (sinusoidal or rectangular) of the visual elements displayed on the screen.

Considering the SSVEP detection methods, the results, obtained by applying each of the three proposed methods to the four types of visual paradigms, showed a superiority of FBCCA over its other two contenders. This is an expected result, consistent with that reported by Ruiz-Olaya et al. (2019) and Liu et al. (2020) when comparing these same three detection methods.

It is noteworthy that as the stimulation frequency increases, the accuracy and ITR parameters decrease. In particular, the frequency of 10,909 Hz indicates maximum values of accuracy. This would be in contradiction with the previous statement since lower values should then be reported with respect to the frequency of 8.57 Hz. However, the explanation may be given by the fact that the stimulation frequency is coincidentally in the center of the alpha band spectrum. In terms of detection time, the CBR paradigm shows the lowest overall values for the stimulus frequencies 8.57 and 10.909 Hz in the three detection methods, and then, for the remaining stimulation frequencies (15, 20, and 24 Hz), the global maximum values of detection times are reached. We found no reasonable explanation for this behavior of detection times other than the influence of artifacts on the EEG data, the presence of alpha and beta activity, and a greater incidence of harmonics as the frequency increases. The consequence of this is that the ITR is maximum for the 8.57 Hz stimulation frequency in each of the three detection methods for the CBR paradigm because the detection time is minimal compared to the other visual paradigms and the accuracy rate is approximately similar. However, for the 10, 909 Hz frequency, the ITR experiences a decrease for the specific cases in which the MEC and CCA methods are applied in the CBR paradigm since, although the detection times remain at the minimum values compared to the ratings associated with the other visual paradigms, the accuracy decreases and consequently the ITR decreases. For the frequencies of 15, 20, and 24 Hz the detection times reach the global maximum values with the CBR paradigm, and at the same time, with the increase of the stimulation frequencies, the accuracy decreases (Regan, 1989; Pathiranage et al., 2018), even below the chance level at 20 and 24 Hz, which is why the ITR becomes null for the MEC detection method, which also applies to the CBS paradigm. Based on the results obtained, we can summarize that the FBCCA method outperforms the other two detection methods and that the best overall performances are obtained by combining this SSVEP detection method with the OOR or OOS paradigms.

In summary, the results suggest that the choice of visual stimuli paradigm can significantly impact the accuracy and ITR parameters of SSVEP detection and that some paradigms may be more effective than others depending on the specific detection method being used. Also, the detection time results suggest that the choice of visual stimuli paradigm may not significantly impact the speed of SSVEP detection. However, it is important to note that this analysis only assessed differences in average DT across the different paradigms and methods. Further analyses or *post-hoc* tests may be needed to investigate whether there are more subtle differences in DT that were not captured by this initial analysis.

Finally, the survey applied to evaluate the participants' comfort is not conclusive since the paradigms involving the checkerboard pattern (CBR and CBS) narrowly outperform those involving the On-Off type (OOR and OOS). However, the fact that the checkerboard pattern is preferred by users, to avoid visual fatigue and improve their focus, is fully in line with previous research reports mentioned next. The observation that 14 of 27 subjects selected checkerboard pattern stimulation as promoting greater visual comfort and focus is consistent with the contrasting explanations provided in several studies. On one hand, this result is aligned with previous publications such as in Duszyk et al. (2014), where it is postulated that visual stimuli that induce stronger SSVEP responses tend to generate greater visual fatigue (OOR and OOS). Additionally, Zheng et al. (2020) discusses how contrast changes in these visual paradigms can be somewhat intense compared to other visual stimuli such as the checkerboard pattern, leading to an increased demand for attention, which in turn derives in the occurrence of visual fatigue. However, as reported in Zerafa et al. (2013) and Choi et al. (2019b), the application of the checkerboard pattern elicits greater discomfort when compared to the On-Off pattern. As a result of these opposing viewpoints in the literature and the minimal prevalence of one visual paradigm over the other in our survey results, we reaffirm that our findings are consistent with this divided opinion on the subject matter and we suggest expanding the population sample in future experiments.

To achieve a balance between performance and comfort we propose the use of the OOS visual paradigm combined with the FBCCA in future instances, but with the modification that the applied stimulation frequencies be in the range of 15–24 Hz. This can be achieved by implementing the Joint Frequency-Phase Modulation (JFPM) method described in Chen et al. (2015b), thus allowing improved discriminability between SSVEPs responses over a narrow range of stimulation frequencies. We consider that the fact that the checkerboard paradigm is more propitious to achieve better visual focus and comfort, is not a strong enough argument to propose this visual pattern in future experiments considering that it outperforms the On-Off paradigm by only one participant. Another element that supports our proposal is the weak performance, manifested in accuracy and ITR values, obtained with this paradigm, mainly for frequencies higher than 15 Hz.

## 5. Conclusions

This study proposes a comprehensive analysis of the effect generated on the performance of an SSVEP-based BCI by simultaneously evaluating different visual stimulation paradigms and detection methods. In summary, and based on the results obtained, we can formulate the following statements: (*i*) On average, the signal-to-noise ratio is higher when an On-Off standard visual stimuli is modulated by sinusoidal (OOS) or rectangular (OOR) signals. This is particularly evident in the occipital channels; (*ii*) evidence indicates that stimulation with oscillating patterns modulated by sinusoidal or rectangular signals and standard scheme (OOS or OOR), when combined with the FBCCA method, leads to better performance; (*iii*) results also suggest that to achieve better performance, the frequencies of the visual stimuli should be between 8.5 and 15 Hz; (*iv*) however, this result is in contrast with the user's perception of comfort, since, according to the survey applied, the checkerboard stimulus pattern, whether modulated by a pulsed or sinusoidal signal (CBR or CBS) and at high frequencies (>20 Hz), favors a more pleasant visual experience and a lower incidence of ocular fatigue.

Our study suggests that there are combinations of visual paradigms with SSVEP detection methods that yield better performance in discriminating targets from non-target items with a pertinent level of confidence.

These results, including the ITR reports, inspire further research exploring stimulation schemes with mixed visual paradigms for specific frequencies implemented in the front-end application. Also, in our study, the superiority of the FBCCA method over MEC and CCA is evident, and therefore its comparison with other successful SSVEP detection methods such as Task-Related Component Analysis (TRCA), under the same visual stimulation variants, would be appropriate.

We provided a free and open-access database of electroencephalographic recordings of the response to four visual paradigms inducing the SSVEP phenomenon. The usefulness of this dataset is oriented to the implementation of new detection algorithms based on the SSVEP paradigm, mainly for a reduced number of elements to be detected, which is common in BCI applications, for example for navigation or remote control of mobility systems, such as robots or wheelchairs.

## Data availability statement

The raw data supporting the conclusions of this article will be made available by the authors, without undue reservation.

## Ethics statement

The studies involving human participants were reviewed and approved by School of Medicine's Ethics Committee in Investigation at Tecnologico de Monterrey (ITESM) and the School of Medicine's Committee of Investigation at Tecnologico de Monterrey (ITESM). The patients/participants provided their written informed consent to participate in this study.

## Author contributions

JC, JA, RC, and OM-M: conceptualization. LH-R, JA, RC, and OM-M: methodology. OM-M: software. JC: conducted the experiments. JC, LH-R, JA, and OM-M: validation and data curation. LH-R, JA, and OM-M: resources. JC, LH-R, JA, RC, and OM-M: writing—original draft preparation. All authors contributed to the article and approved the submitted version.
