# Supplementary material for: SSVEP detection assessment by combining visual stimuli paradigms and no-training detection methods
Source: Front Neurosci. 2023 May 18;17:1142892. doi: 10.3389/fnins.2023.1142892 (PMC10233154; doi:10.3389/fnins.2023.1142892)
Supplement: Supplementary file 1 [file Data_Sheet_1.pdf]

Supplementary Material

1 SUPPLEMENTARY FIGURES

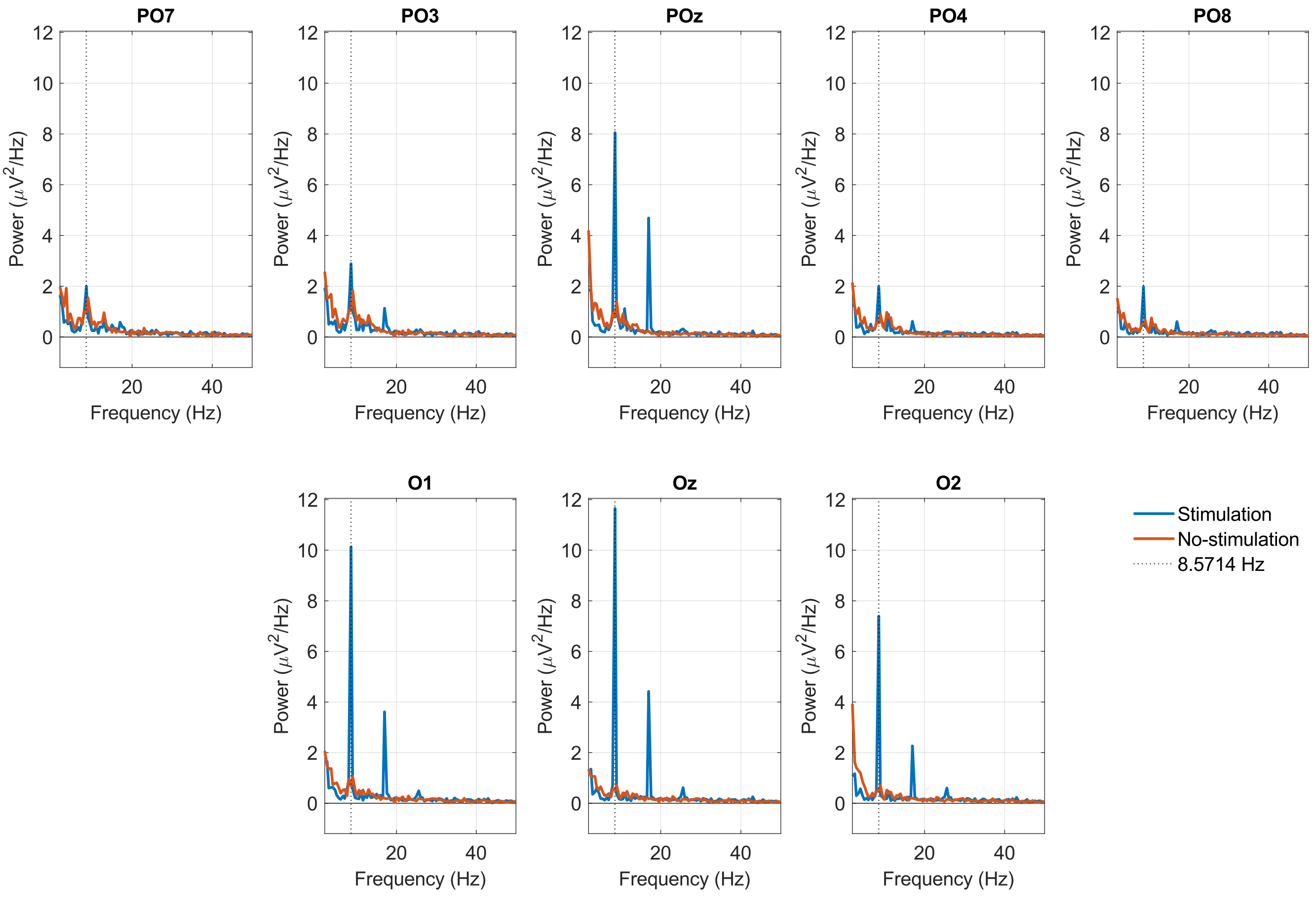

Figure S1. Average EEG power response per channel for the stimulation frequency of $8.57Hz$ when the rectangular modulated On-Off (OOR) pattern was applied to subject 27.

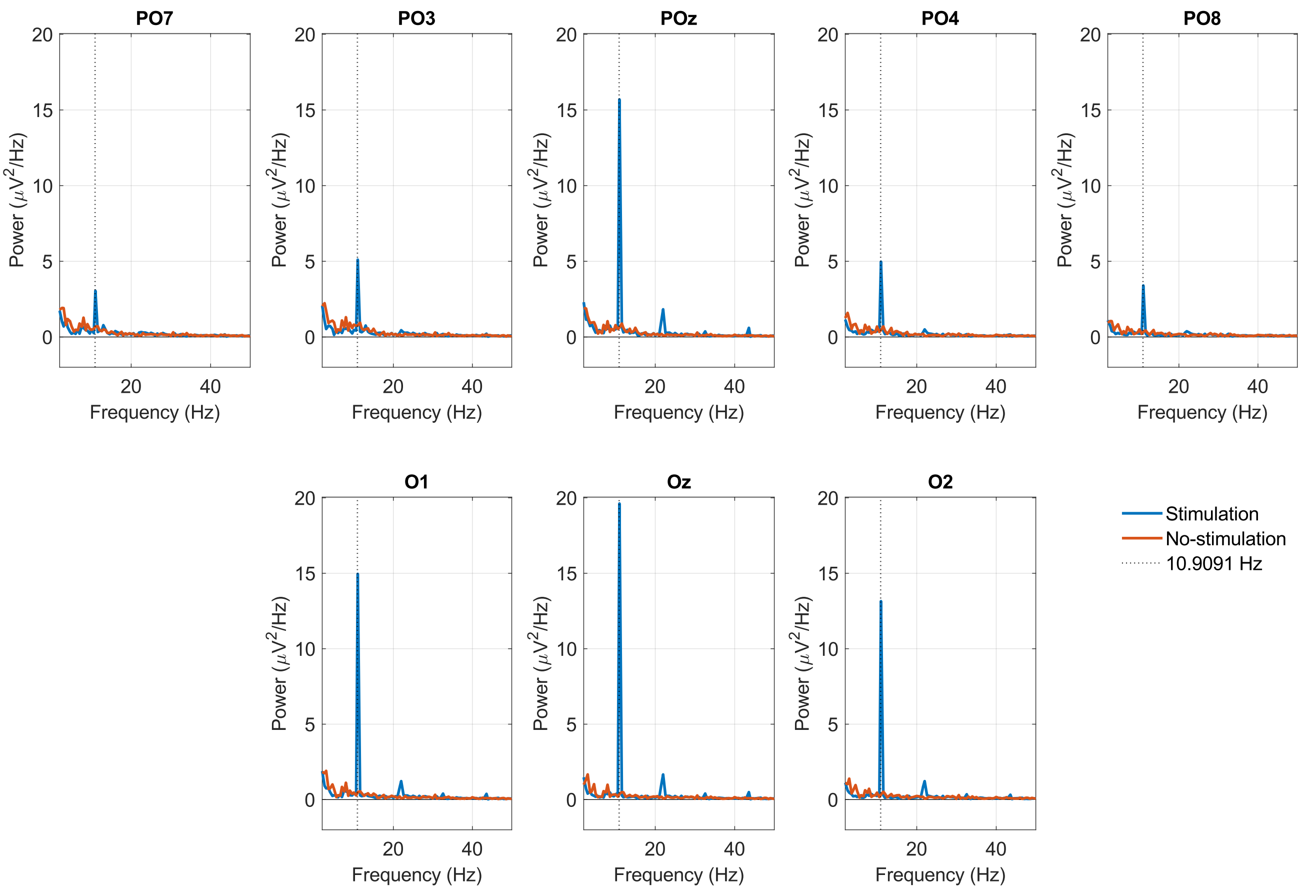

Figure S2. Average EEG power response per channel for the stimulation frequency of $10.909Hz$ when the rectangular modulated On-Off (OOR) pattern was applied to subject 27.

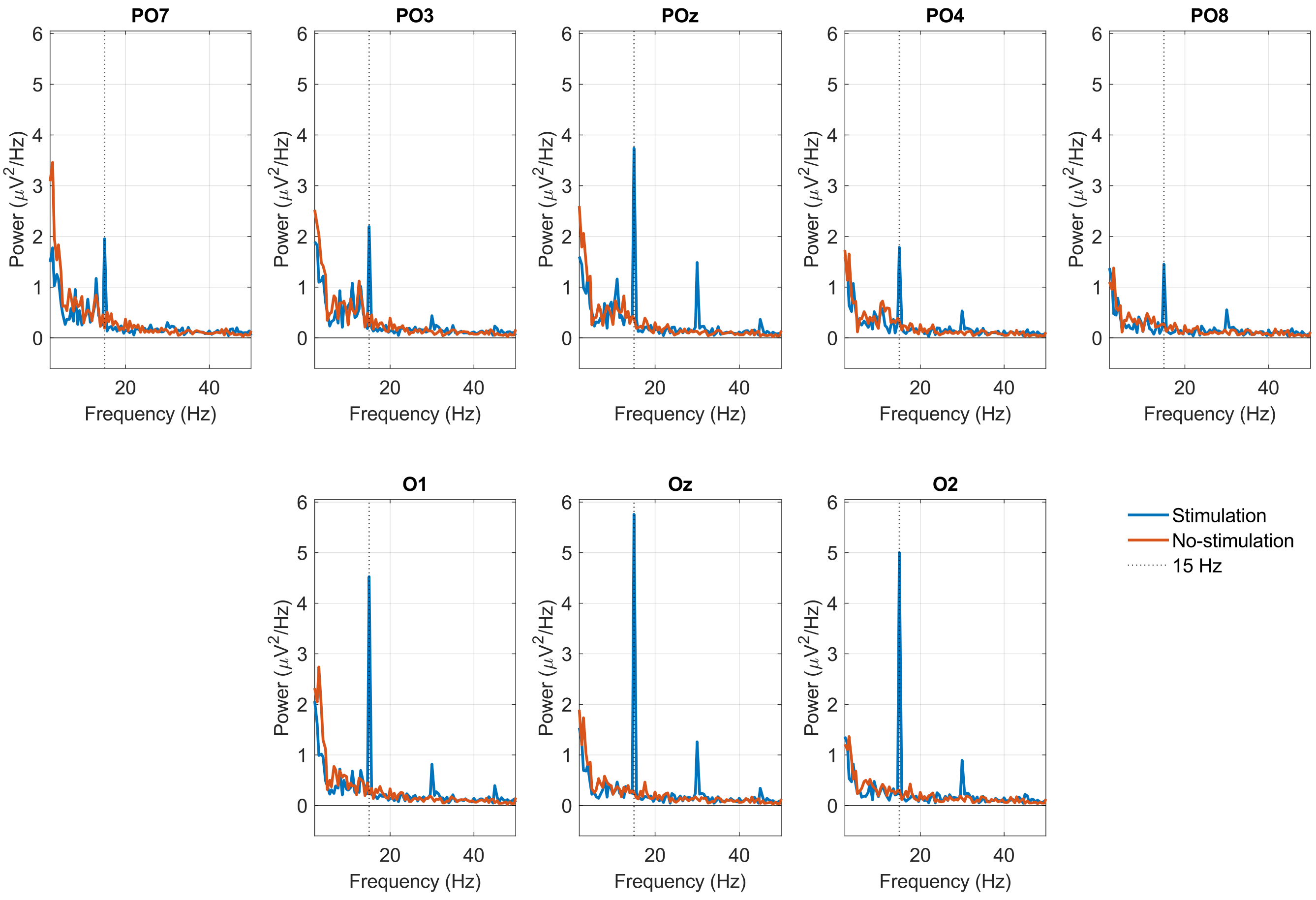

Figure S3. Average EEG power response per channel for the stimulation frequency of $15Hz$ when the rectangular modulated On-Off (OOR) pattern was applied to subject 27.

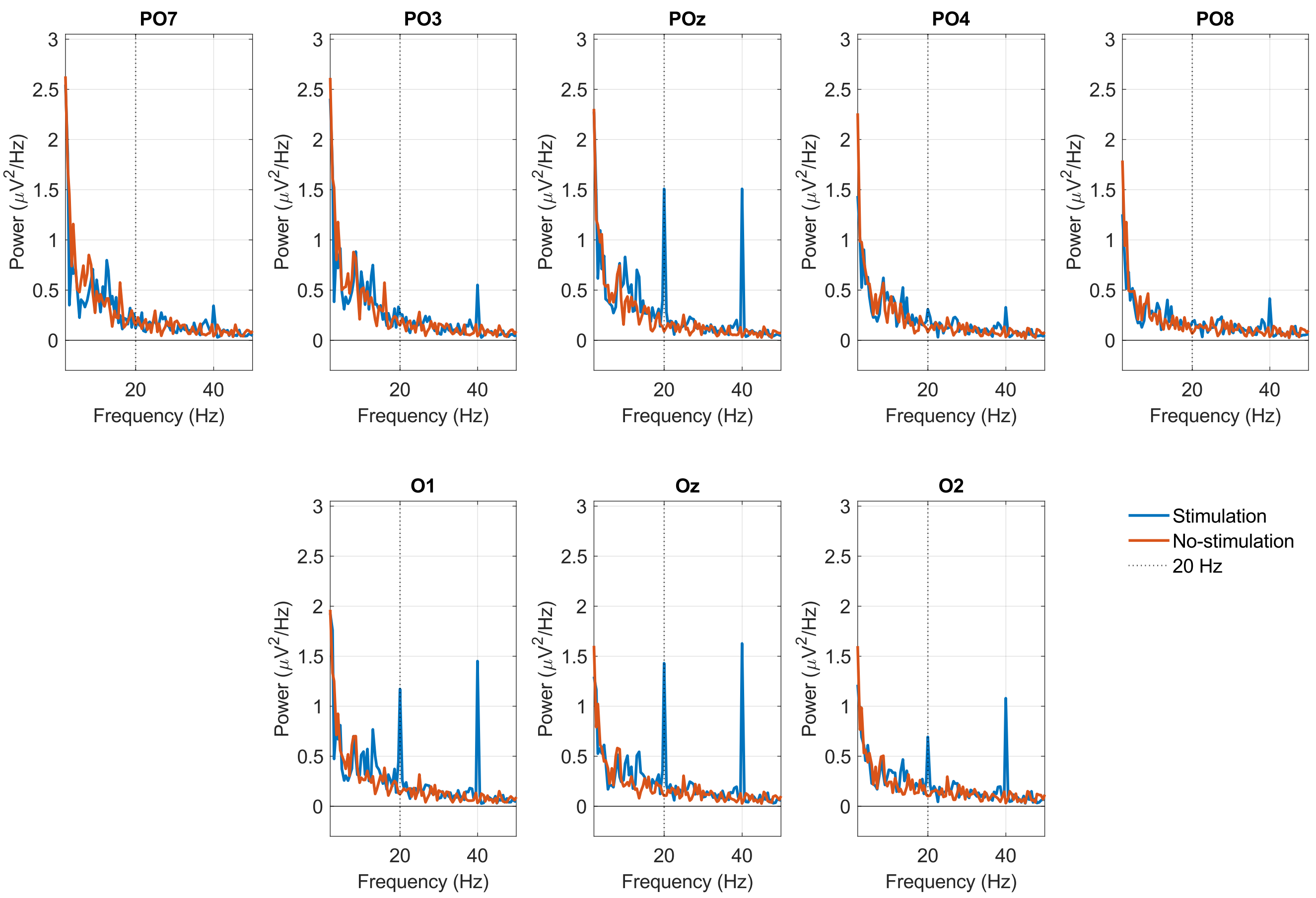

Figure S4. Average EEG power response per channel for the stimulation frequency of $20Hz$ when the rectangular modulated On-Off (OOR) pattern was applied to subject 27.

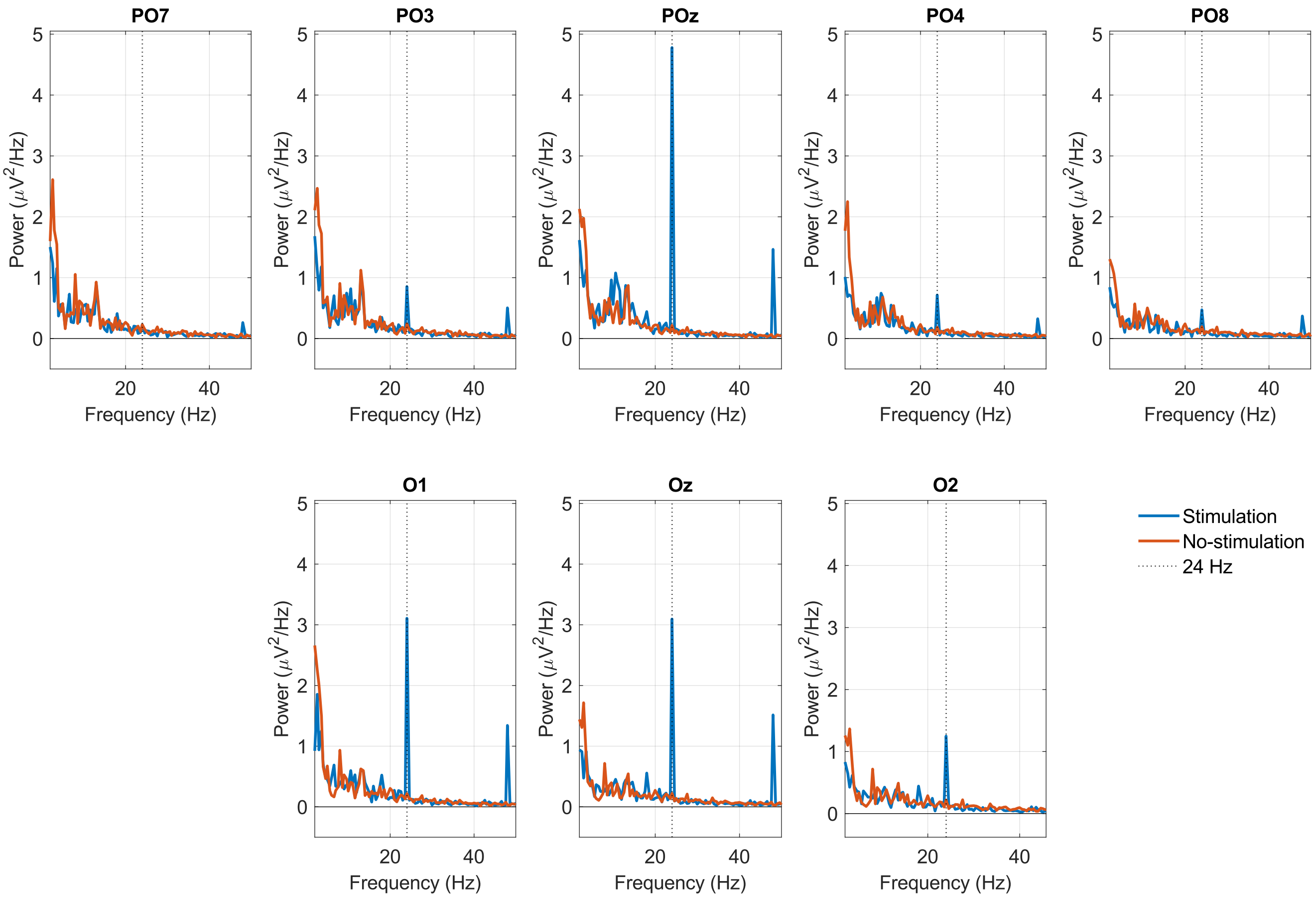

Figure S5. Average EEG power response per channel for the stimulation frequency of $24Hz$ when the rectangular modulated On-Off (OOR) pattern was applied to subject 27.

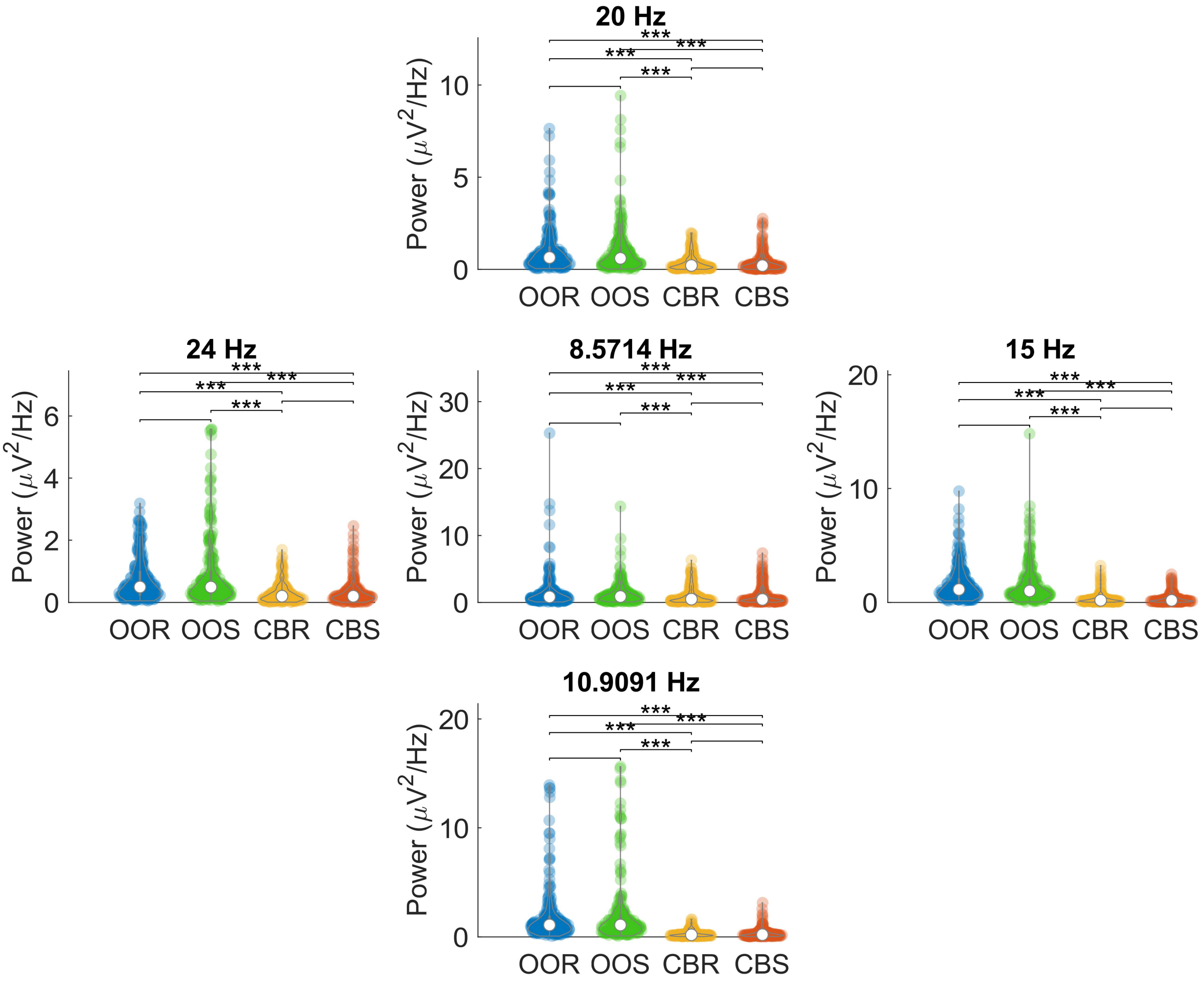

Figure S6. Violin-plot representation of power distribution across participants and across channels for each stimulation frequency ($f_1 : 8.57Hz$; $f_2 : 10.909Hz$; $f_3 : 15Hz$; $f_4 : 20Hz$ and $f_5 : 24Hz$) and each visual paradigm applied (CBR, CBS, OOS, and OOR). Significant differences between pairs of groups of visual paradigms are represented with asterisks, such that $***$ represents $p\_value <= 0.001$; no asterisk above the bracket means ”not significant”.

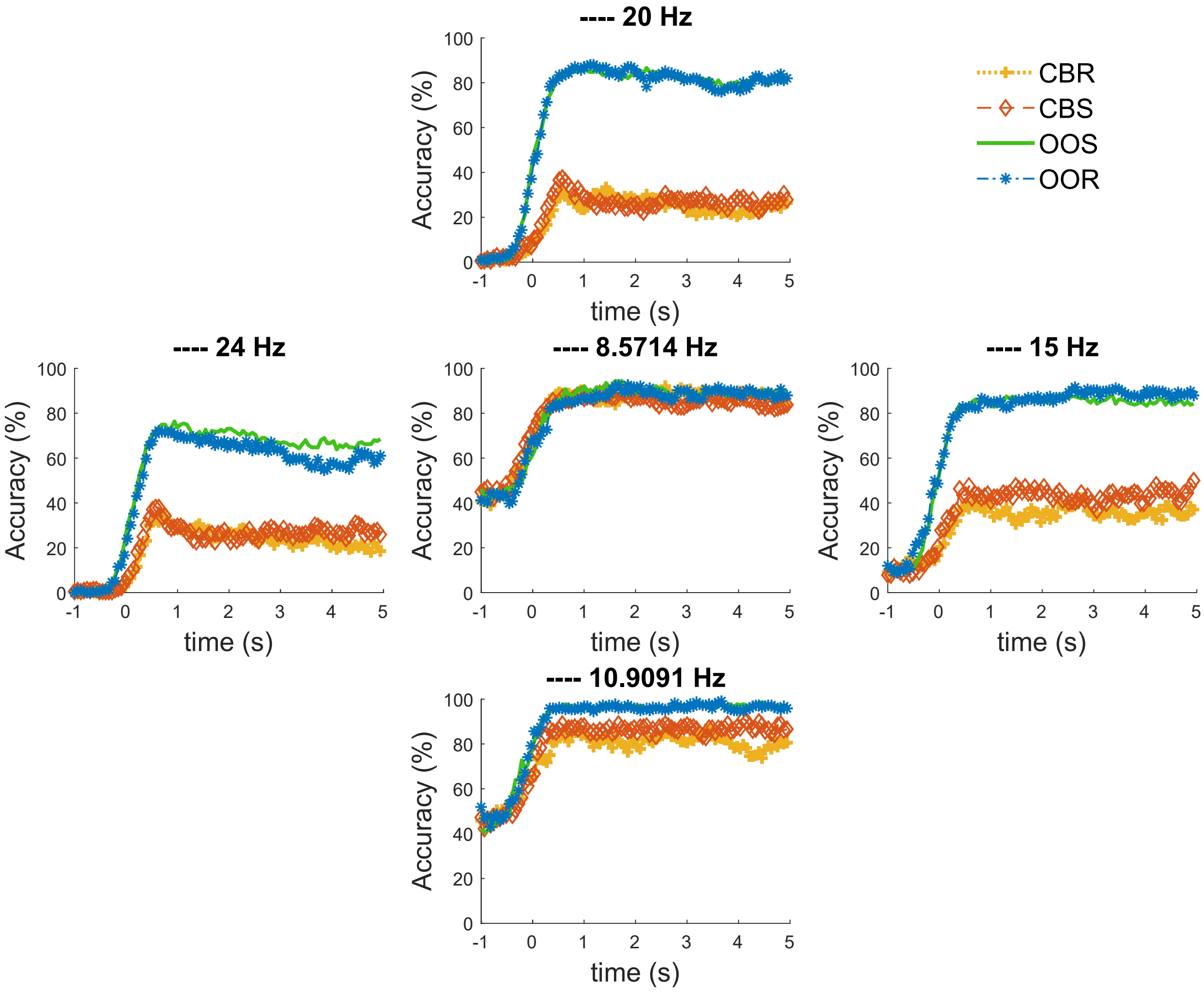

---- 20 Hz
---- 24 Hz
---- 8.5714 Hz
---- 15 Hz
---- 10.9091 Hz
Accuracy (%)
time (s)
CBR
CBS
OOS
OOR

(a)

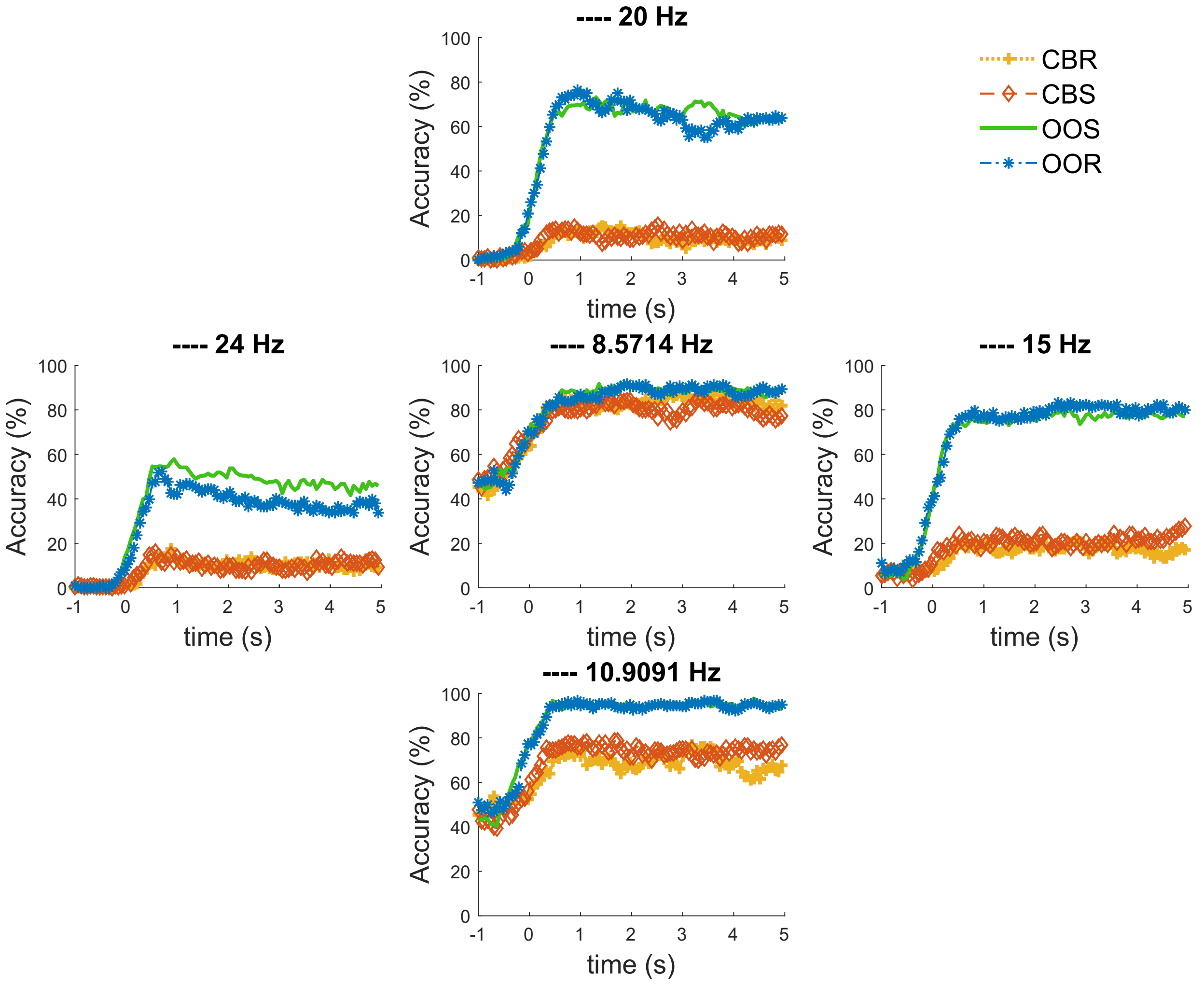
---- 20 Hz
---- 24 Hz
---- 8.5714 Hz
---- 15 Hz
---- 10.9091 Hz
Accuracy (%)
time (s)
CBR
CBS
OOS
OOR

(b)

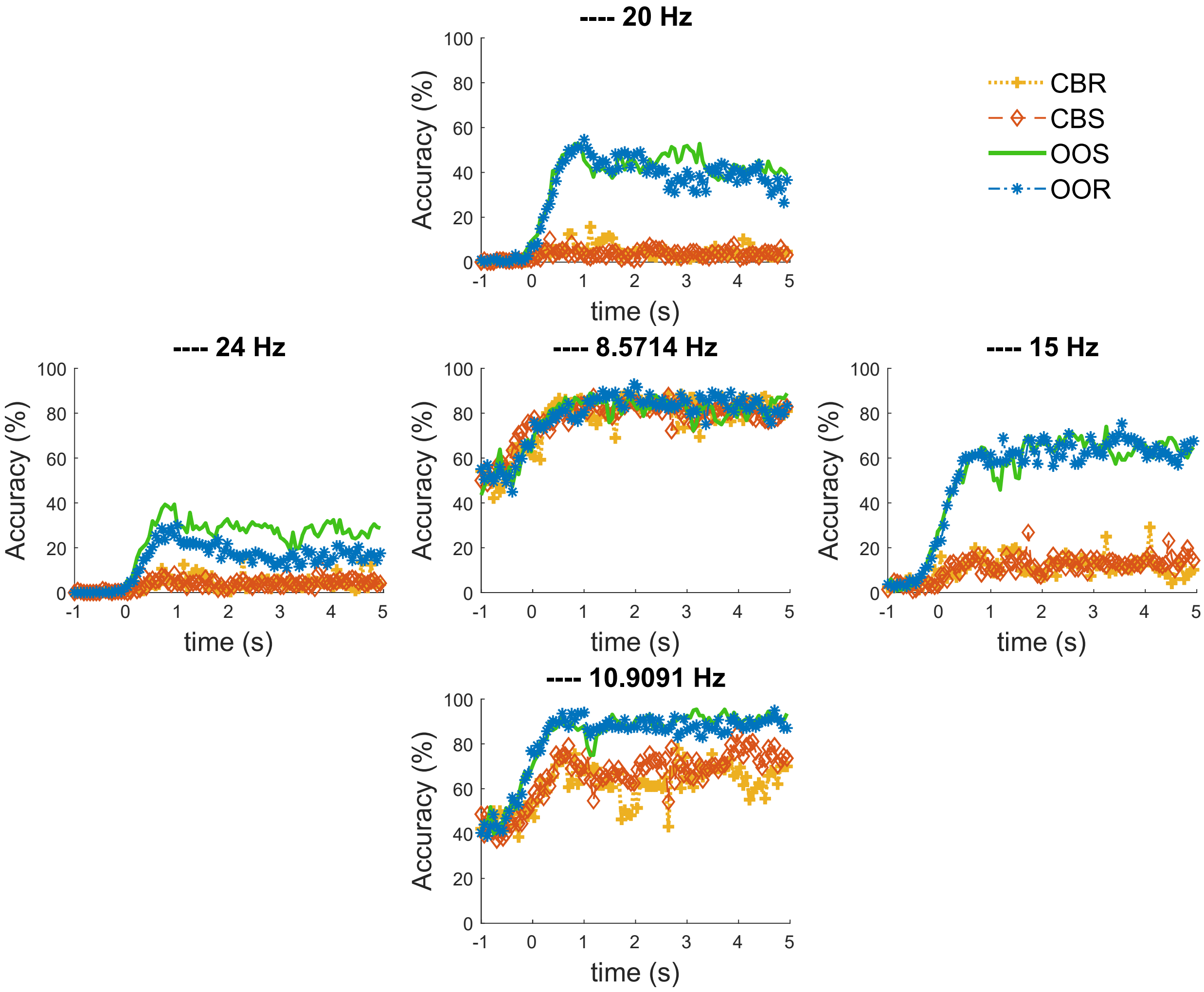

(c)

Figure S7. Across participants, runs and trials Detection Accuracy (DA) versus time, for each stimulation frequency ($f_1 : 8.57Hz$; $f_2 : 10.909Hz$; $f_3 : 15Hz$; $f_4 : 20Hz$ and $f_5 : 24Hz$) and each visual paradigm applied (CBR, CBS, OOS, and OOR): (a) FBCCA (b) CCA (c) MEC

2 SUPPLEMENTARY TABLES

Table S1. Across participants and trials Wide-band SNR per channel, stimulation frequency, and visual paradigm, considering $N_h = 4$, where N_h is the number of harmonics.

	f1				*f2*				*f3*				*f4*				*f5*			
	**OOR**	**OOS**	**CBR**	**CBS**	**OOR**	**OOS**	**CBR**	**CBS**	**OOR**	**OOS**	**CBR**	**CBS**	**OOR**	**OOS**	**CBR**	**CBS**	**OOR**	**OOS**	**CBR**	**CBS**
**PO7**	**-10.57**	-11.65	-13.12	-12.46	-12.17	**-11.84**	-13.49	-15.44	-14.19	**-12.80**	-16.53	-16.86	-15.52	**-15.39**	-18.01	-18.18	-16.18	**-16.03**	-19.60	-19.19
**PO3**	**-10.80**	-11.35	-11.70	-11.98	-11.21	**-11.02**	-13.27	-13.50	-12.80	**-12.04**	-16.63	-16.25	-15.01	**-14.97**	-17.57	-17.40	-15.55	**-14.86**	-18.91	-18.31
**POZ**	-9.72	**-9.53**	-10.78	-10.91	-8.76	**-8.41**	-12.44	-12.70	-10.10	**-9.65**	-15.68	-15.31	-12.29	**-11.99**	-16.50	-16.43	-13.15	**-11.93**	-17.28	-16.78
**PO4**	-10.73	**-10.66**	-10.55	-10.89	-9.80	**-9.62**	-11.93	-12.89	**-10.60**	-11.50	-15.41	-15.53	-12.78	**-12.31**	-16.18	-16.54	-14.05	**-13.30**	-17.39	-17.24
**PO8**	-12.23	**-11.86**	-14.55	-11.99	**-11.96**	-12.97	-15.35	-13.66	**-11.94**	-13.74	-18.48	-16.65	-14.62	**-13.70**	-19.63	-16.72	**-15.38**	-15.98	-19.24	-18.54
**O1**	-8.74	**-8.70**	-10.21	-10.21	-8.58	**-8.10**	-12.90	-13.06	-9.52	**-9.31**	-14.66	-15.33	-12.09	**-11.93**	-15.74	-15.59	-13.22	**-12.32**	-16.72	-16.57
**OZ**	-7.03	**-6.49**	-8.58	-8.40	-6.48	**-5.85**	-11.66	-12.18	-7.01	**-6.76**	-13.18	-13.50	-9.11	**-8.90**	-13.78	-13.59	-10.74	**-9.31**	-14.55	-14.70
**O2**	-8.57	**-8.06**	-9.03	-9.07	-7.86	**-7.06**	-12.00	-12.19	-9.25	**-7.96**	-14.15	-14.45	-10.89	**-10.47**	-14.34	-14.51	-12.11	**-10.89**	-15.37	-15.91
